# Self-propagating, protease-resistant, recombinant prion protein conformers with or without *in vivo* pathogenicity

**DOI:** 10.1371/journal.ppat.1006491

**Published:** 2017-07-12

**Authors:** Fei Wang, Xinhe Wang, Christina D. Orrú, Bradley R. Groveman, Krystyna Surewicz, Romany Abskharon, Morikazu Imamura, Takashi Yokoyama, Yong-Sun Kim, Kayla J. Vander Stel, Kumar Sinniah, Suzette A. Priola, Witold K. Surewicz, Byron Caughey, Jiyan Ma

**Affiliations:** 1 Center for Neurodegenerative Science, Van Andel Research Institute, Grand Rapids, Michigan, United States of America; 2 Rocky Mountain Laboratories, National Institute of Allergy & Infectious Diseases, National Institutes of Health, Hamilton, Montana, United States of America; 3 Department of Physiology and Biophysics, Case Western Reserve University, Cleveland, Ohio, United States of America; 4 National Institute of Oceanography and Fisheries (NIOF), Cairo, Egypt; 5 National Institute of Animal Health, National Agriculture and Food Research Organization (NARO), Tsukuba, Ibaraki, Japan; 6 Ilsong Institute of Life Science, Korea CJD Diagnostic Center, Hallym University, Anyang, Republic of Korea; 7 Department of Chemistry and Biochemistry, Calvin College, Grand Rapids, Michigan, United States of America; University of Alberta, CANADA

## Abstract

Prions, characterized by self-propagating protease-resistant prion protein (PrP) conformations, are agents causing prion disease. Recent studies generated several such self-propagating protease-resistant recombinant PrP (rPrP-res) conformers. While some cause prion disease, others fail to induce any pathology. Here we showed that although distinctly different, the pathogenic and non-pathogenic rPrP-res conformers were similarly recognized by a group of conformational antibodies against prions and shared a similar guanidine hydrochloride denaturation profile, suggesting a similar overall architecture. Interestingly, two independently generated non-pathogenic rPrP-res were almost identical, indicating that the particular rPrP-res resulted from cofactor-guided PrP misfolding, rather than stochastic PrP aggregation. Consistent with the notion that cofactors influence rPrP-res conformation, the propagation of all rPrP-res formed with phosphatidylglycerol/RNA was cofactor-dependent, which is different from rPrP-res generated with a single cofactor, phosphatidylethanolamine. Unexpectedly, despite the dramatic difference in disease-causing capability, RT-QuIC assays detected large increases in seeding activity in both pathogenic and non-pathogenic rPrP-res inoculated mice, indicating that the non-pathogenic rPrP-res is not completely inert *in vivo*. Together, our study supported a role of cofactors in guiding PrP misfolding, indicated that relatively small structural features determine rPrP-res’ pathogenicity, and revealed that the *in vivo* seeding ability of rPrP-res does not necessarily result in pathogenicity.

## Author summary

Many neurodegenerative disorders, including Alzheimer’s disease, Parkinson’s disease and Prion disease, are caused by misfolded proteins that can self-propagate *in vivo* and *in vitro*. Misfolded self-replicating recombinant prion protein (PrP) conformers have been generated *in vitro* with defined cofactors, some of which are highly infectious and cause bona fide prion diseases, while others completely fail to induce any pathology. Here we compare these misfolded recombinant PrP conformers and show that the non-pathogenic misfolded recombinant PrP is not completely inert *in vivo*. We also found that the pathogenic and non-pathogenic recombinant PrP conformers share a similar overall architecture. Importantly, our study clearly shows that *in vivo* seeded spread of misfolded conformation does not necessarily lead to pathogenic change or cause disease. These findings not only are important for understanding the molecular basis for prion infectivity, but also may have important implications for the “prion-like” spread of misfolded proteins in other neurodegenerative diseases.

## Introduction

Transmissible spongiform encephalopathies (TSEs), also known as prion diseases, are a group of fatal neurodegenerative disorders affecting both humans and other mammals[1]. A central pathogenic event in prion disease is conformational conversion of the host-encoded prion protein (PrP^C^), a normal, protease-sensitive, cell-surface localized glycoprotein, to a misfolded and protease-resistant pathogenic conformer, PrP^Sc^[1–5]. As an unorthodox infectious agent, PrP^Sc^ replicates itself by imprinting its distinctive infectious conformation on host PrP^C^ molecules[6]. The molecular mechanisms underlying the *in vivo* PrP^C^-to-PrP^Sc^ conversion are largely unknown. Recent *in vitro* studies have revealed that bacterially expressed recombinant PrP (rPrP) can be converted into pathogenic conformations in a test tube and those pathogenic forms cause *bona fide* prion disease in animals[7–13]. Even though some *in vitro* rPrP conversions in the absence of any additives have produced prion infectivity[11–13], generation of rPrP pathogenic conformers with a proper, i.e. scrapie-like, proteinase K (PK)-resistant pattern and a high titer of prion infectivity have so far been achieved only in the presence of cofactors[7–10].

The serial protein misfolding cyclic amplification (sPMCA) is one of the methods commonly used to study prion conversion *in vitro*[14,15]. During sPMCA, a mixture of PrP^C^-containing normal brain homogenate plus a small amount of PrP^Sc^-containing diseased brain homogenate is subject to successive cycles of sonication and incubation, allowing simultaneous propagation of the PrP^Sc^ conformers and prion infectivity[16]. The enormous amplification power makes sPMCA a sensitive tool for detecting minute amounts of PrP^Sc^, and this methodology has been successfully used for the diagnosis of prion disease[17,18].

The sPMCA can also be performed without any seed, allowing *de novo* generation of PrP^Sc^. Using the latter approach, we have shown that, in the presence of total RNA isolated from mouse liver plus synthetic phospholipid POPG (1-palmitoyl-2-oleoyl-*sn*-glycero-3-phospho-(1′-*rac*-glycerol), recombinant murine prion protein (rPrP) purified from *E*. *coli* can be converted into the highly infectious and PK-resistant conformer rPrP-res^RNA^[7,19], which causes prion disease in wild-type animals and has the same pathogenic properties as naturally occurring prions[8]. A similar conversion system, containing rPrP, total mouse liver RNA, and POPG, was used at the NIH Rocky Mountain Laboratory to produce an rPrP-res conformer (rPrP-res^NIH^) *de novo*[20]. Interestingly, despite its ability to propagate indefinitely by sPMCA, rPrP-res^NIH^ does not cause disease *in vivo*[20].

In sPMCA reactions seeded by native murine prions or rPrP-res^RNA^, phosphatidylethanolamine (PE) was found to allow rPrP conversion into the PK-resistant and highly pathogenic conformer rPrP-res^PE^ as the only cofactor[10]. Omitting the PE cofactor resulted in either the halting of rPrP-res^PE^ propagation or the generation of a cofactor-independent, “protein-only”, PK-resistant conformer rPrP-res^protein-only^ that failed to induce any pathology in mice[21]. The non-pathogenic rPrP-res^protein-only^ has a smaller PK-resistant core than rPrP-res^PE^, but nonetheless can be propagated indefinitely by sPMCA[21]. The fact that the non-pathogenic rPrP-res^protein-only^ could be propagated in the absence of any cofactor raised a series of interesting questions. Is the lack of infectivity in certain non-pathogenic rPrP-res conformers due to the absence of cofactors? Are cofactors required for the propagation of other non-pathogenic rPrP-res conformers? Do all non-pathogenic rPrP-res conformers share the same structure? And how significant is the difference between the structures of the non-pathogenic and pathogenic rPrP-res conformers?

Here we performed detailed comparisons between the highly pathogenic rPrP-res^RNA^ and two independently formed, non-pathogenic rPrP-res conformers in terms of their propagation, PK-resistance patterns, conformational differences, capability of infecting cultured cells, and seeding of rPrP amyloid fibril growth. Our results provided novel insights into the relationship among cofactors, self-propagating conformations, and *bona fide* prion infectivity.

## Results

### *De novo* formed rPrP-res^RNA-low^ does not contain detectable *in vivo* pathogenicity

**Fig 1:**
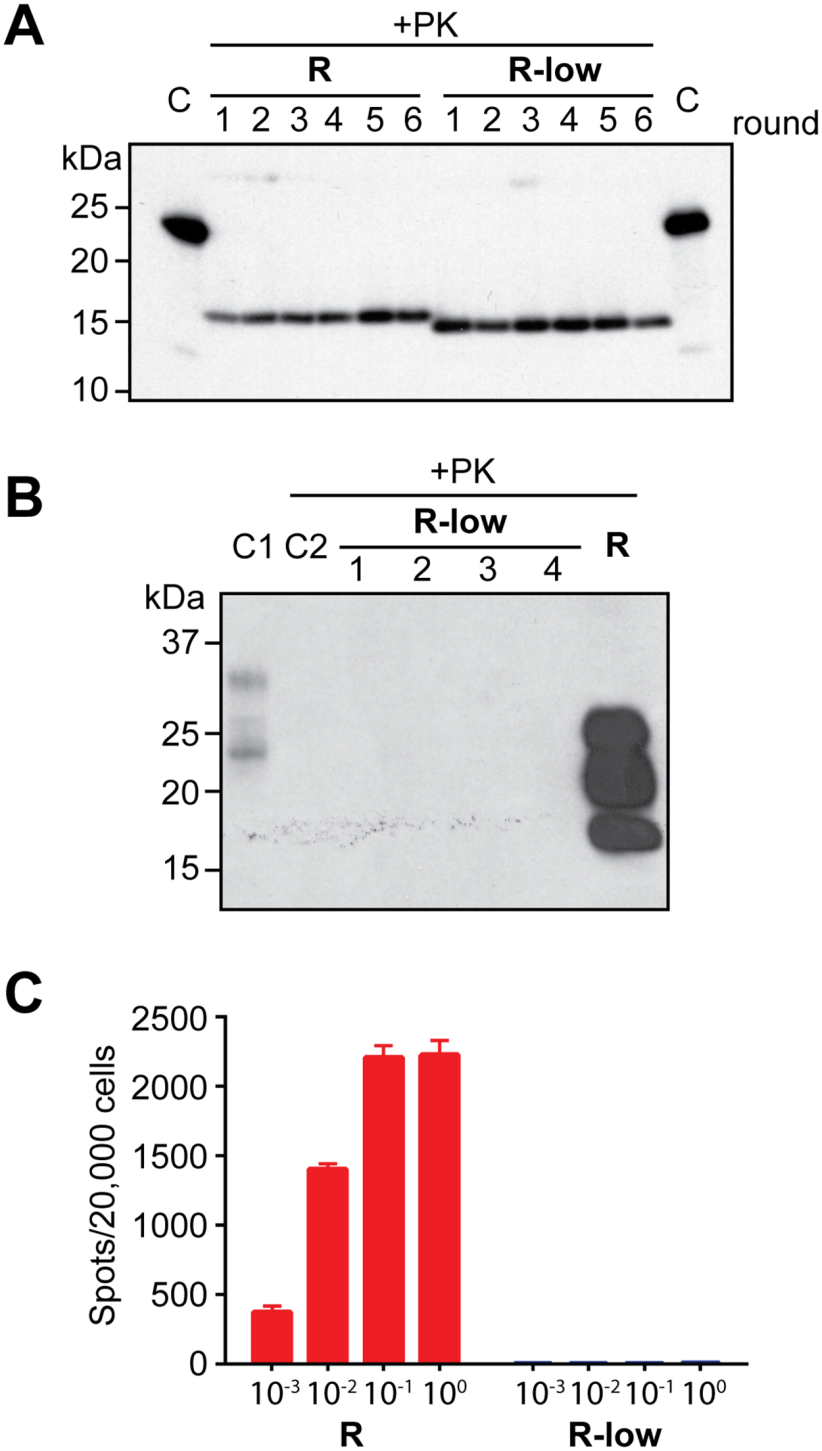
*De novo* formed self-propagating rPrP-res^RNA-low^ lacks *in vivo* pathogenicity. (**A**) Both rPrP-res^RNA^ (R) and rPrP-res^RNA-low^ (R-low) propagate indefinitely in the presence of RNA and POPG cofactors. C: undigested rPrP as controls. (**B**) Brain homogenates of 4 mice inoculated with rPrP-res^RNA-low^ (R-low), 1 representative mouse inoculated with rPrP-res^RNA^ (R) in bioassay experiment #1 (Table 1), and 1 healthy uninfected control mouse (C2) were digested with PK, and PK-resistant PrP fragments were detected by western blotting with M20 polyclonal anti-PrP antibody. C1: brain homogenate of a healthy uninfected control mouse. (**C**) The infectivity of rPrP-res^RNA^ (R) and rPrP-res^RNA-low^ (R-low) was evaluated by the Elispot cell infection assay.

We previously reported that in the presence of total mouse liver RNA and synthetic POPG, purified and fully folded rPrP can be converted into highly pathogenic recombinant prion (rPrP-res^RNA^) in an unseeded sPMCA reaction[7]. Using the same protocol, we found that another rPrP-res form, rPrP-res^RNA-low^, could also be generated *de novo* (S1 Fig). The rPrP-res^RNA-low^ has a smaller PK-resistant core (about 15 kDa, vs. ~16 kDa for rPrP-res^RNA^). Similar to the pathogenic rPrP-res^RNA^, rPrP-res^RNA-low^ can propagate indefinitely by sPMCA (Fig 1A).

To determine whether rPrP-res^RNA-low^ is pathogenic *in vivo*, we intracerebrally inoculated wild-type C57BL/6 mice with rPrP-res^RNA^ or rPrP-res^RNA-low^. Consistent with our previous report[7], mice inoculated with rPrP-res^RNA^ developed prion disease after a relatively synchronized incubation period and presented histopathological changes in the brain typical of prion disease (Table 1). In animals inoculated with rPrP-res^RNA-low^, multiple animal bioassays (including secondary transmission in wild-type C57BL/6 mice or intracerebral inoculation in Tga20 transgenic mice that overexpress wild-type PrP^C^ (Table 1)) failed to show any signs of prion disease, or any pathology. Brain homogenates from rPrP-res^RNA-low^ mice sacrificed at 477–583 dpi (days post injection) were subjected to PK digestion and no PK-resistant PrP was detected in those mice (Fig 1B). The lack of pathology was also confirmed by histopathological analyses (S2–S5 Figs).

**Table 1:** Mouse bioassay of rPrP-res^RNA-low^.

	Inoculum	Mouse strain	Route	Diseased / injected	Survival time (dpi)
Experiment #1 (passage 1)	rPrP-res^RNA^	C57BL/6	Intracerebral inoculation	5/5	170, 170, 172, 173, 176
	rPrP-res^RNA-low^ (Batch #1)	C57BL/6	Intracerebral inoculation	0/5	583, 583, 583, 477^a^, 399^b^^,^^c^
Experiment #1 (passage 2)	Mouse brain homogenate	C57BL/6	Intracerebral inoculation	0/5	504, 504, 504, 504, 419^d^
Experiment #2	rPrP-res^RNA-low^ (Batch #2)	C57BL/6	Intracerebral inoculation	0/5	499, 499, 499, 499, 499
Experiment #3	rPrP-res^RNA-low^ (Batch #3)	Tga20	Intracerebral inoculation	0/4	500, 500, 500, 500

^a^ This mouse died of an intercurrent disease and no PK-resistant PrP^Sc^ was detected in the brain.

^b^ This mouse was healthy when sacrificed at 399 dpi to evaluate the potential histopathological changes in the brain.

^c^ Brain homogenate of this mouse was used to inject a group of 5 mice for the second passage.

^d^ This mouse died of an intercurrent disease.

Compared to the traditional rodent bioassay, the Elispot-based cultured-cell prion infection assay is a sensitive, more rapid and economical assay[22,23]. Because of the restrictive sensitivity of cultured cells to prion strains, we chose CAD5 cells, which are known to be susceptible to a broad spectrum of murine prion strains[22,23]. When naïve CAD5 cells were infected with either rPrP-res^RNA^ or rPrP-res^RNA-low^, the Elispot data matched very well with traditional animal bioassay results[8], showing a significant amount of prion infectivity from rPrP-res^RNA^, but none from rPrP-res^RNA-low^ (Fig 1C).

Lysates of the CAD5 cells used in the Elispot assay were further verified by classic PK digestion and western blots, confirming that rPrP-res^RNA^ efficiently converted endogenous PrP^C^ into PrP^Sc^, but rPrP-res^RNA-low^ failed to infect CAD5 cells (S6 Fig). Together, these results confirmed that the self-propagating rPrP-res^RNA-low^ does not cause any pathology *in vivo*, nor does it contain any detectable prion infectivity in cell culture assay. Moreover, these data suggest that the co-existence of a self-propagating PK-resistant rPrP conformation and cofactors does not automatically produce prion infectivity.

### Cofactors are required for propagating both rPrP-res^RNA^ and rPrP-res^RNA-low^

**Fig 2:**
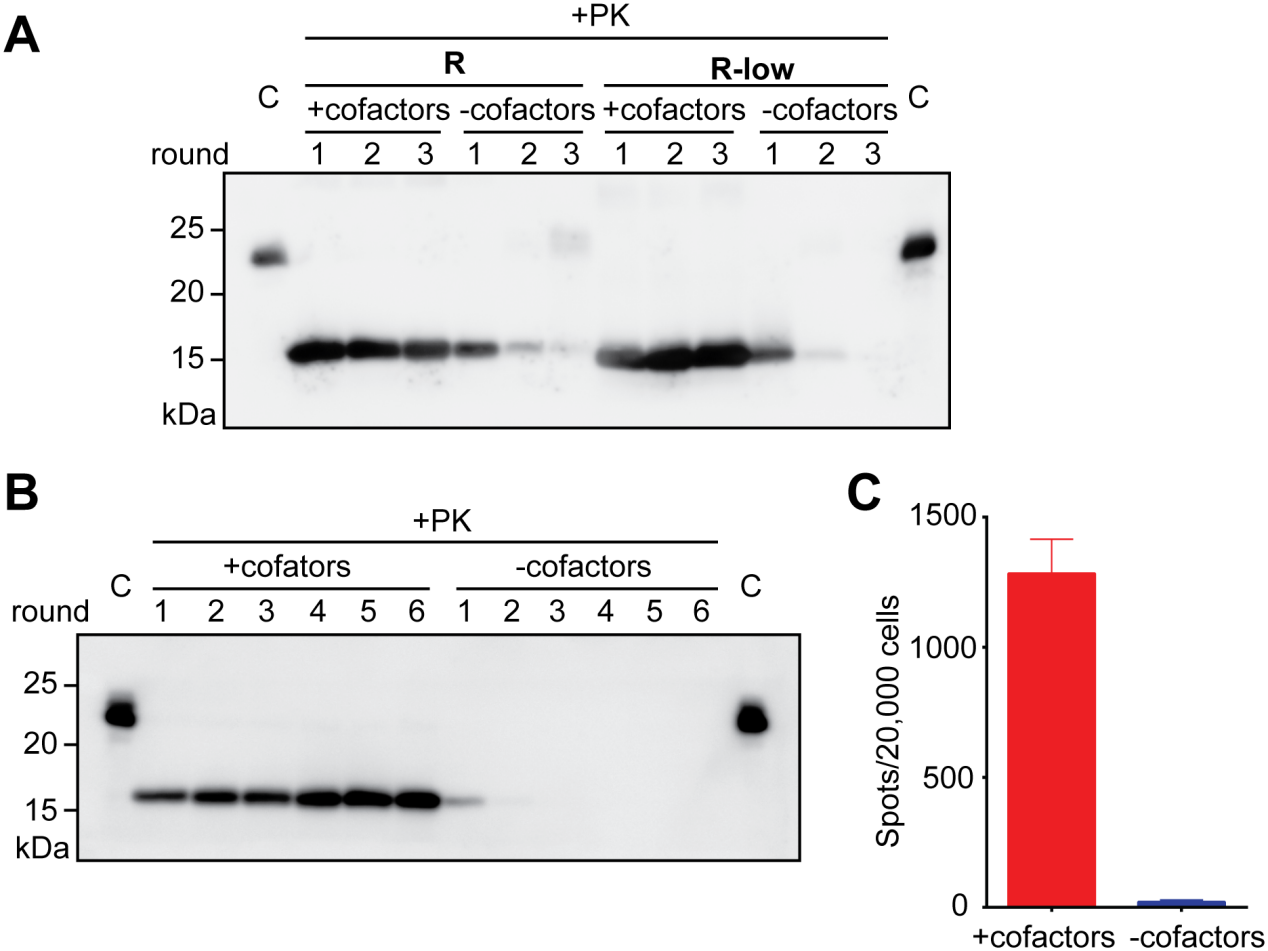
The propagation of both pathogenic rPrP-res^RNA^ and non-pathogenic rPrP-res^RNA-low^ is cofactor-dependent. (**A**) In seeded sPMCA reactions, rPrP-res^RNA^ (R) or rPrP-res^RNA-low^ (R-low) were added to complete substrates (+cofactors) and to substrates without any cofactor (-cofactors). The mixtures were subjected to 3 rounds of sPMCA as indicated. After each round, 10 μL of PMCA product was collected and subjected to PK digestion, SDS-PAGE, and western blotting. C: undigested rPrP as controls. (**B**) Six rounds of sPMCA seeded by rPrP-res^RNA^ with (+cofactors) or without cofactors (-cofactors). After each round, 10 μL of PMCA product was collected and subjected to PK digestion, SDS-PAGE, and western blotting. C: rPrP as controls. (**C**) The Elispot cell infection assay of round-6 sPMCA products from panel **B**.

Since the non-pathogenic rPrP-res^protein-only^ is able to replicate without any cofactor[21], we tested whether the propagation of rPrP-res^RNA-low^ was cofactor-dependent. The sPMCA was carried out either with substrate lacking any cofactor and containing only rPrP or with the complete substrate, i.e., rPrP plus cofactors. In the presence of cofactors, both pathogenic rPrP-res^RNA^ and non-pathogenic rPrP-res^RNA-low^ were propagated efficiently, but in the absence of cofactors, neither of the conformers sustained their propagation (Fig 2A). The requirement of cofactors for rPrP-res^RNA-low^ propagation suggests that even though rPrP-res^RNA-low^ shares with rPrP-res^protein-only^ the properties of being non-pathogenic and a smaller PK-resistant core, it is different from the cofactor-independent rPrP-res^protein-only^.

Since the lack of PK-resistant PrP does not always correlate with loss of prion infectivity[24], we determined whether the failure of rPrP conversion correlated with a loss of infectivity by the Elispot infection assay. The rPrP-res^RNA^ was used to seed sPMCA reactions with either complete substrate or substrate lacking the cofactor for 6 rounds to ensure that no residual infectivity was carried over from the infectious rPrP-res^RNA^ seed. As expected, the rPrP-res conformer was only generated in the presence of cofactors, but not in the cofactor-free reactions (Fig 2B). The 6th-round sPMCA products were used to infect naïve CAD5 cells, and the Elispot assay revealed that the prion infectivity had been propagated along with rPrP-res^RNA^ in regular sPMCA, but no prion infectivity was detected in sPMCA performed in the absence of cofactors (Fig 2C and S7 Fig). These results support an intimate association between the self-propagating rPrP-res conformations and prion infectivity.

### Biochemical and morphological properties of rPrP-res^RNA^ and rPrP-res^RNA-low^

**Fig 3:**
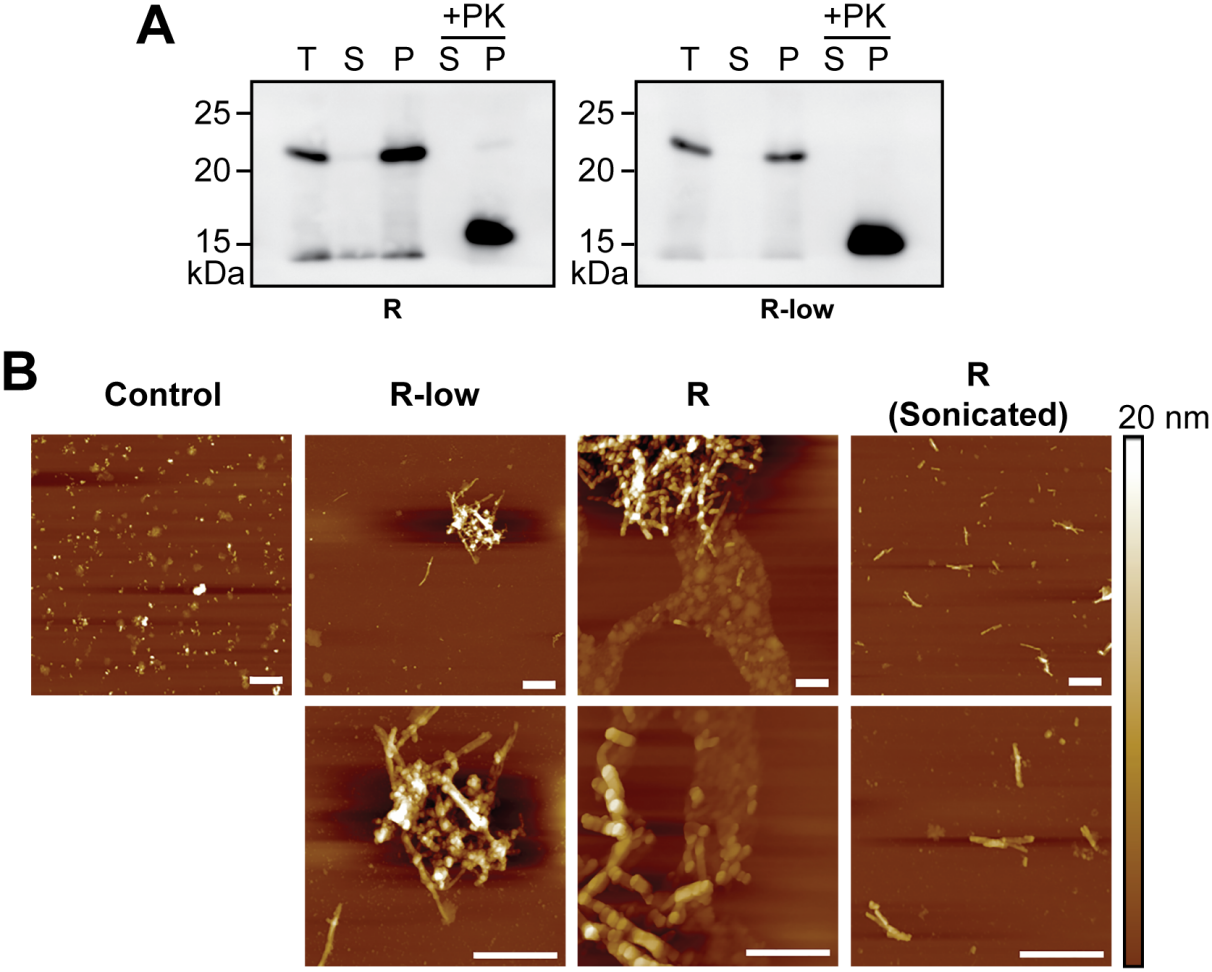
Biochemical and morphological properties of rPrP-res^RNA^ and rPrP-res^RNA-low^. (**A**) Two hundred microliters of sPMCA products of rPrP-res^RNA^ (R) and rPrP-res^RNA-low^ (R-low) were centrifuged at 100,000 x *g* at 4°C for 1 h. The supernatant was collected and the pellet was resuspended in 200 μL of PMCA buffer. For the total (T), supernatant (S), and pellet (P) fractions, 1 μL of each fraction was loaded onto SDS-PAGE; for PK digested samples, 10 μL of S or P was subjected to PK digestion followed by SDS-PAGE and western blotting. (**B**) Representative atomic force microscopy images of different rPrP-res aggregates. Control sample contains cofactors only in the absence of rPrP. The sample was processed in identical way as those containing rPrP. Upper panels are images of Control, rPrP-res^RNA-low^ (R-low), rPrP-res^RNA^ (R) and sonicated rPrP-res^RNA^ (R (Sonicated)) at lower magnification. Bottom panels are images of rPrP-res^RNA-low^ (R-low), rPrP-res^RNA^ (R) and sonicated rPrP-res^RNA^ (R (Sonicated)) at higher magnification. Bars at each panel represent 400 nm.

Although rPrP-res^RNA^ and rPrP-res^RNA-low^ were propagated under exactly the same conditions—i.e., using the same batch of rPrP, the same batch of cofactors, and the same sPMCA parameters—these two rPrP-res conformers differed drastically in their biological activities. This difference led us to ask whether these two conformers differed in any of their other properties besides the obvious size difference in their PK-resistant cores. We found that both rPrP-res^RNA^ and rPrP-res^RNA-low^ appeared in the pellet fraction after ultracentrifugation (Fig 3A), suggesting that both were aggregated. Analysis of these aggregates by atomic force microscopy (AFM) revealed the presence of relatively short fibrillar structures that clumped together forming large aggregates (Fig 3B, R and R-low), and became more apparent when large clumps were partially dispersed by sonication (Fig 3B, R (sonicated)). No obvious morphological differences could be detected by AFM between rPrP-res^RNA^ and rPrP-res^RNA-low^ aggregates. It should be noted that fibrillar clumps were accompanied by nonfibrillar particles of varied morphologies (in some fields imaged by AFM only the latter structures were seen). However, similar particles were also present in control sPMCA samples containing cofactors in the absence of rPrP (Fig 3B, control), making it difficult to conclude whether such nonfibrillar particles correspond to rPrP-containing structures or those formed by cofactors only.

**Fig 4:**
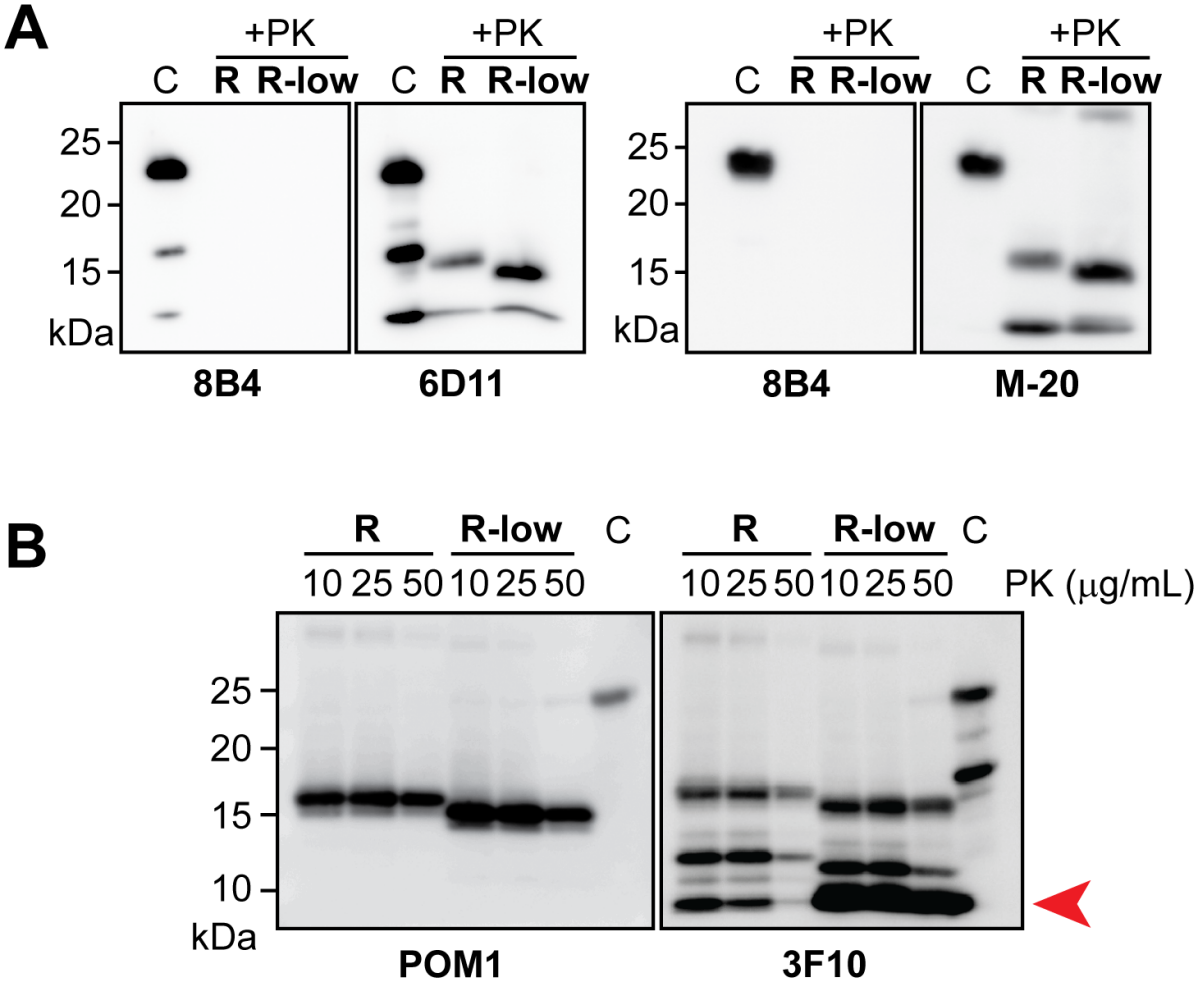
Biochemical analyses of rPrP-res^RNA^ and rPrP-res^RNA-low^. (**A**) Ten microliters of rPrP-res^RNA^ (R) and rPrP-res^RNA-low^ (R-low) were PK-digested and detected by immunoblot analysis with 8B4 (monoclonal antibody against an N-terminal epitope at residue 35–45), 6D11 (monoclonal antibody against the epitope at residues 93–109), or M-20 (polyclonal antibody against 90–230) anti-PrP antibodies as indicated. (**B**) Serial PK digestions of rPrP-res^RNA^ (R) and rPrP-res^RNA-low^ (R-low). Western blots were first probed with POM1 (against C-terminal conformational epitopes) then reprobed with 3F10 (against the epitope at residues 137–151) anti-PrP monoclonal antibodies.

Western blots probed with anti-PrP antibodies, i.e., monoclonal 6D11 (recognizing an epitope of 93–109 of PrP) and polyclonal M20 (recognizing PrP90-230), showed that the PK-resistant core of rPrP-res^RNA-low^ was a C-terminal fragment similar to that of rPrP-res^RNA^ (Fig 4A). Notably, the size difference between the cores remained relatively constant following digestions with increasing amounts of PK (Fig 4B, POM1), indicating that both rPrP-res forms have relatively stable PK-resistant cores. The monoclonal 3F10 antibody, recognizing an epitope encompassing residues 137–151 of PrP[25], detected more PK-resistant fragments on the same blot (Fig 4B, 3F10). The banding patterns were consistent and distinct for rPrP-res^RNA^ and rPrP-res^RNA-low^, particularly the stronger small bands of rPrP-res^RNA-low^ at the forefront of the gel (Fig 4B, 3F10, arrow), confirming the structural difference between those two rPrP-res conformers.

**Fig 5:**
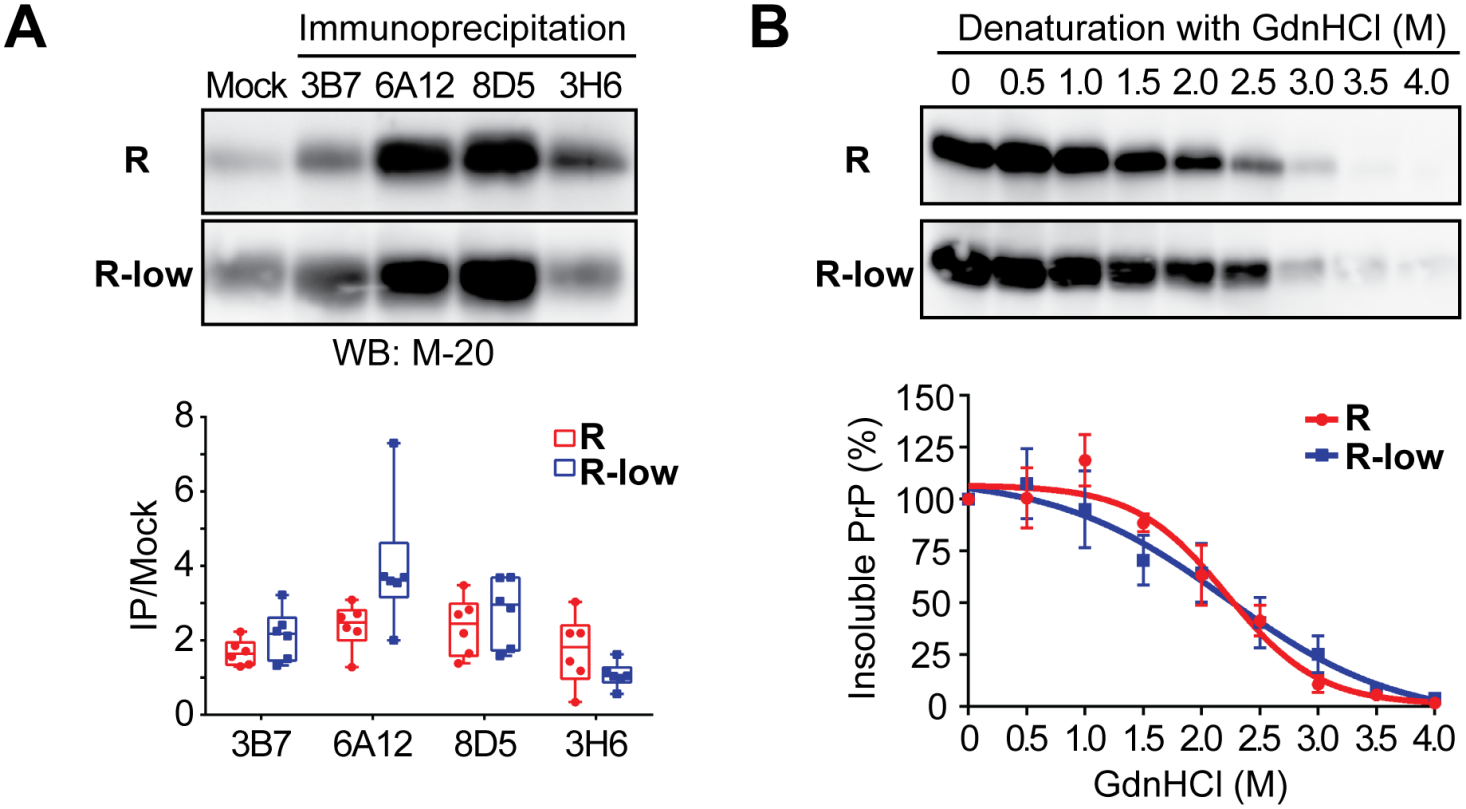
rPrP-res^RNA^ and rPrP-res^RNA-low^ share a similar architecture. (**A**) Representative western blots of immunoprecipitation analysis for rPrP-res^RNA^ (R) and rPrP-res^RNA-low^ (R-low) using a panel of anti-PrP^Sc^ conformational antibodies: 3B7, 6A12, 8D5, and 3H6. The 6A12 and 8D5 antibodies are against N-terminal epitopes; the epitopes for 3B7 and 3H6 are unclear. Immunoprecipitated PrP was detected by western blotting using M-20 polyclonal anti-PrP antibody (left panel). Data from 6 immunoprecipitation analyses are summarized in the Box and Whisker plot (right panel). Statistical evaluation was performed with multiple t tests using the Holm-Sidak method in GraphPad Prism, which revealed that there is no significant difference (*p*> 0.05) between rPrP-res^RNA^ (R) and rPrP-res^RNA-low^ (R-low). (**B**) Representative western blots (upper panel) of conformational stability assay and denaturation curves (lower panel) for rPrP-res^RNA^ (R) and rPrP-res^RNA-low^ (R-low). Statistical evaluation was performed with two-way ANOVA followed by Tukey’s multiple comparisons test in GraphPad Prism, which revealed that there is no significant difference between rPrP-res^RNA^ (R) and rPrP-res^RNA-low^ (R-low).

To further probe the conformational differences between rPrP-res^RNA^ and rPrP-res^RNA-low^, we performed an immunoprecipitation assay with a panel of four conformational antibodies that were raised to specifically capture PrP^Sc^ molecules[26,27]. Interestingly, none of the antibodies was able to unambiguously differentiate rPrP-res^RNA^ from rPrP-res^RNA-low^ (Fig 5A), suggesting that despite the obvious difference in the size of their PK-resistant cores, the pathogenic and non-pathogenic rPrP-res forms share a similar overall architecture. This conclusion was further supported by the guanidine hydrochloride (GdnHCl) denaturation assay (Fig 5B). In this assay, increased concentrations of GdnHCl gradually solubilize aggregated rPrP-res, allowing a differentiation of different prion strains[28]. Notably, when rPrP-res^RNA^ and rPrP-res^RNA-low^ were exposed to increased concentrations of GdnHCl, the [GdnHCl]_1/2_ for the two were 2.22 ± 0.13 and 2.15 ± 0.35, respectively. Moreover, the curves of insoluble rPrP were very similar and no statistical difference could be detected at any GdnHCl concentration (Fig 5B). Thus, both the conformational antibody binding and GdnHCl denaturation assay suggest that rPrP-res^RNA^ from rPrP-res^RNA-low^ share a similar overall architecture.

### The rPrP-res^NIH^ shares the same biochemical properties with rPrP-res^RNA-low^

**Fig 6:**
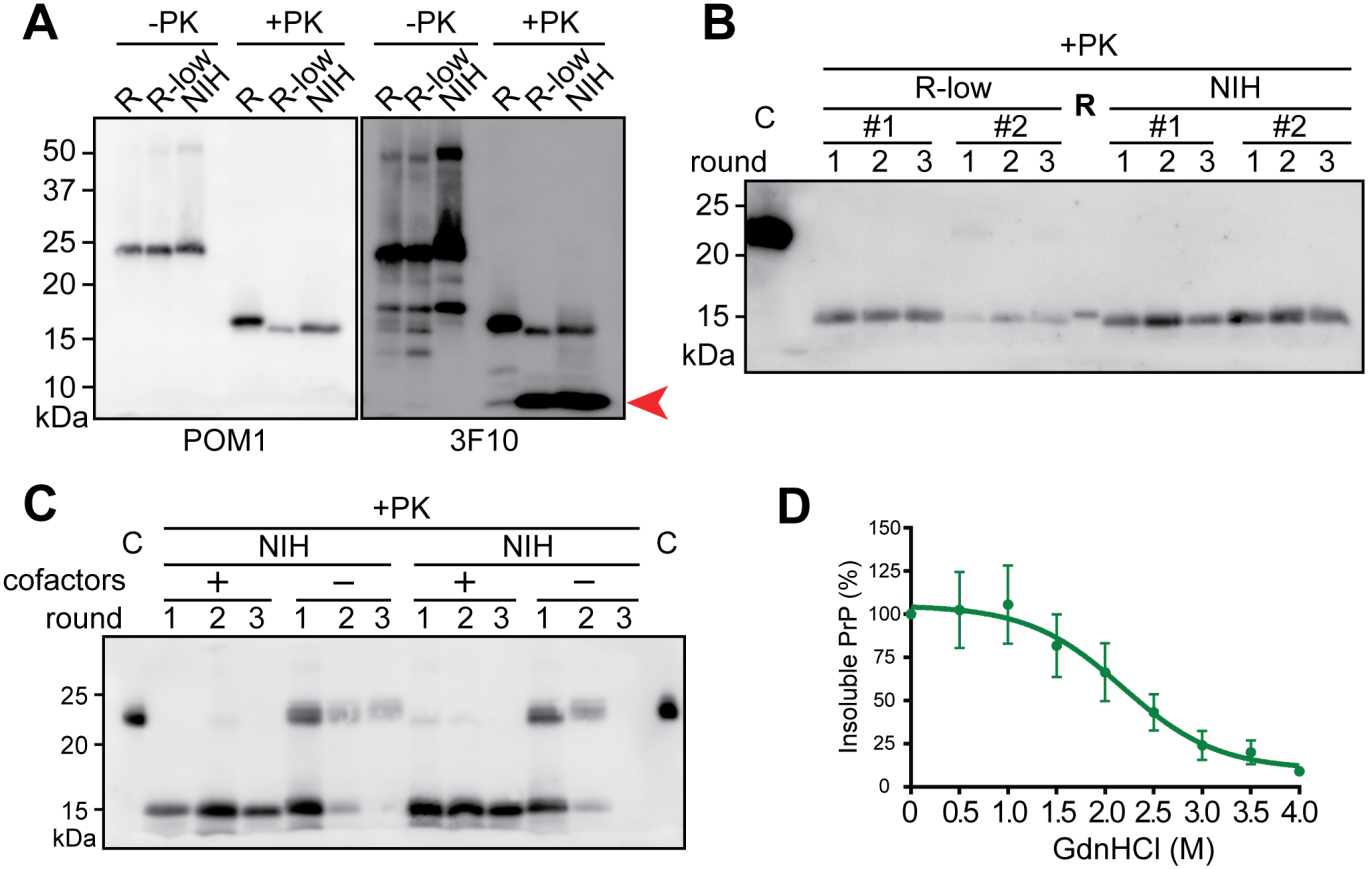
Comparisons between rPrP-res^RNA^, rPrP-res^RNA-low^ and rPrP-res^NIH^. (**A**) rPrP-res^RNA^ (R), rPrP-res^RNA-low^ (R-low) and rPrP-res^NIH^ (NIH) were PK-digested, and PK-resistant PrP fragments were detected by western blotting using POM1 (left panel) and then 3F10 (right panel) anti-PrP monoclonal antibodies. (**B**) Representative sPMCA reactions seeded by rPrP-res^RNA-low^ (R-low) or rPrP-res^NIH^ (NIH). The PK-resistant fragment of rPrP-res^RNA^ (R) and undigested rPrP (C) were used as controls. (**C**) Representative sPMCA reactions seeded by rPrP-res^NIH^ (NIH) with complete (+) or cofactor-free (-) substrates. C, undigested rPrP as controls. (**D**) GdnHCl denaturation curve for rPrP-res^NIH^. Two-way ANOVA analyses followed by Tukey’s multiple comparisons test in GraphPad Prism revealed that there is no significant difference between either rPrP-res^RNA^ (R) and rPrP-res^NIH^ (NIH) or rPrP-res^RNA-low^ (R-low) and rPrP-res^NIH^ (NIH).

Because the pathogenic rPrP-res^RNA^ and non-pathogenic rPrP-res^RNA-low^ were generated in the same lab, we expanded our comparison to another non-pathogenic conformer, rPrP-res^NIH^, which was generated *de novo* independently at the NIH Rocky Mountain Laboratory[20]. The banding patterns of rPrP-res^RNA-low^ and rPrP-res^NIH^ detected by the POM1 and 3F10 antibodies were almost identical, highlighted by the stronger small bands detected by the 3F10 antibody (Fig 6A, arrow). The sPMCA results showed that rPrP-res^NIH^ was able to propagate in a separate laboratory under the same conditions used to generate rPrP-res^RNA^ and rPrP-res^RNA-low^ (Fig 6B). More importantly, the propagation of rPrP-res^NIH^ also depended on the presence of the cofactor molecules (Fig 6C). The Elispot assay using CAD5 cells confirmed that both rPrP-res^RNA-low^ and rPrP-res^NIH^ lacked the capability of converting endogenous PrP^C^ (S8 Fig). The GdnHCl denaturation assay of rPrP-res^NIH^ revealed that the curve of insoluble PrP was similar to that of rPrP-res^RNA^ or rPrP-res^RNA-low^ ([GdnHCl]_1/2_ for rPrP-res^NIH^ was 2.18 ± 0.29) and no statistical difference could be detected at any GdnHCl concentration among three sets of data (Fig 6D and S1 Appendix).

Collectively, our results revealed that despite being generated *de novo* in two independent laboratories, the non-pathogenic rPrP-res^RNA-low^ and rPrP-res^NIH^ resemble each other, suggesting that they represent the same non-pathogenic, self-propagating rPrP-res structure. Thus, the *de novo* rPrP-res formation is likely via a distinct PrP misfolding pathway guided by POPG/RNA cofactors.

### Both pathogenic and non-pathogenic rPrP-res conformers seed rPrP amyloid fibrils growth

**Fig 7:**
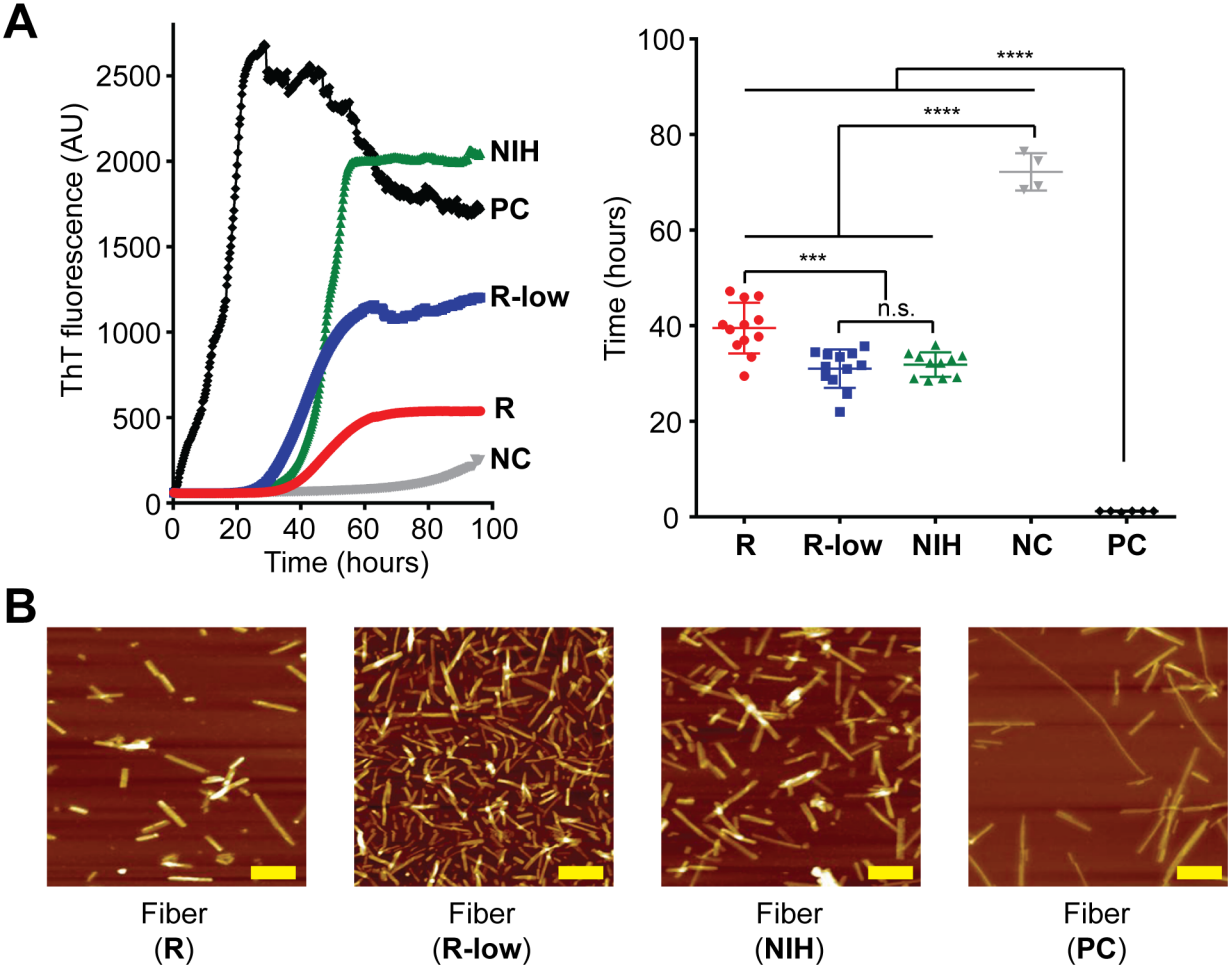
Amyloid fibril seeding abilities of rPrP-res conformers. (**A**) Amyloid fibril growth curves (left panel) and lag times (right panel) for unseeded reaction (NC, n = 4) and reactions seeded by rPrP-res^RNA^ (R, n = 12), rPrP-res^RNA-low^ (R-low, n = 12), rPrP-res^NIH^ (NIH, n = 11), or preformed rPrP fibrils as a positive control (PC, n = 6). n.s., no significance; ***, *p*< 0.001; ****, *p*< 0.0001. Statistical evaluation was performed with ordinary one-way ANOVA (F (4, 40) = 213.5) followed by Tukey’s multiple comparisons test in GraphPad Prism. (**B**) Representative AFM images of fibrils formed with the different seeds. The yellow bar represents 200 nm.

One characteristic of native prions is their ability to seed rPrP amyloid fibril formation[29–31]. Using the semi-denaturing rPrP amyloid fibril formation assay that monitors fibril growth with Thioflavin T (ThT) fluorescence[32], we found that all three rPrP-res conformers—pathogenic rPrP-res^RNA^, non-pathogenic rPrP-res^RNA-low^, and non-pathogenic rPrP-res^NIH^—seeded amyloid fibril formation (Fig 7). The lag phases of reactions seeded by rPrP-res^RNA-low^ and rPrP-res^NIH^ were similar, in both cases, significantly shorter than that of rPrP-res^RNA^-seeded reactions (Fig 7A). Furthermore, the lag phases of all rPrP-res-seeded reactions were significantly longer than reactions seeded by preformed rPrP amyloid fibrils. The amyloid fibrils seeded by all three rPrP-res conformers were morphologically indistinguishable from each other or from those seeded by the preformed rPrP amyloid fibrils (Fig 7B). In addition, the amyloid fibrils seeded by either pathogenic or non-pathogenic rPrP-res conformers showed no infectivity in the Elispot cell infection assay (S9 Fig).

### Detecting *in vitro* and *in vivo* seeding activity of rPrP-res^RNA^ and rPrP-res^RNA-low^ by RT-QuIC

The real-time quaking induced conversion assay (RT-QuIC) is a newly developed prion seeding assay that is also based on the ability of prions to seed rPrP amyloid fibril growth. The RT-QuIC, using a non-denaturing, near-neutral pH reaction system different than the semi-denaturation system for rPrP amyloid fibril growth used in the above experiments, is highly sensitive and has been successfully used to diagnose prion disease in humans and animals[31,33–35]. Using this assay, we compared the *in vitro* and *in vivo* seeding activity of the pathogenic rPrP-res^RNA^ and non-pathogenic rPrP-res^RNA-low^.

**Fig 8:**
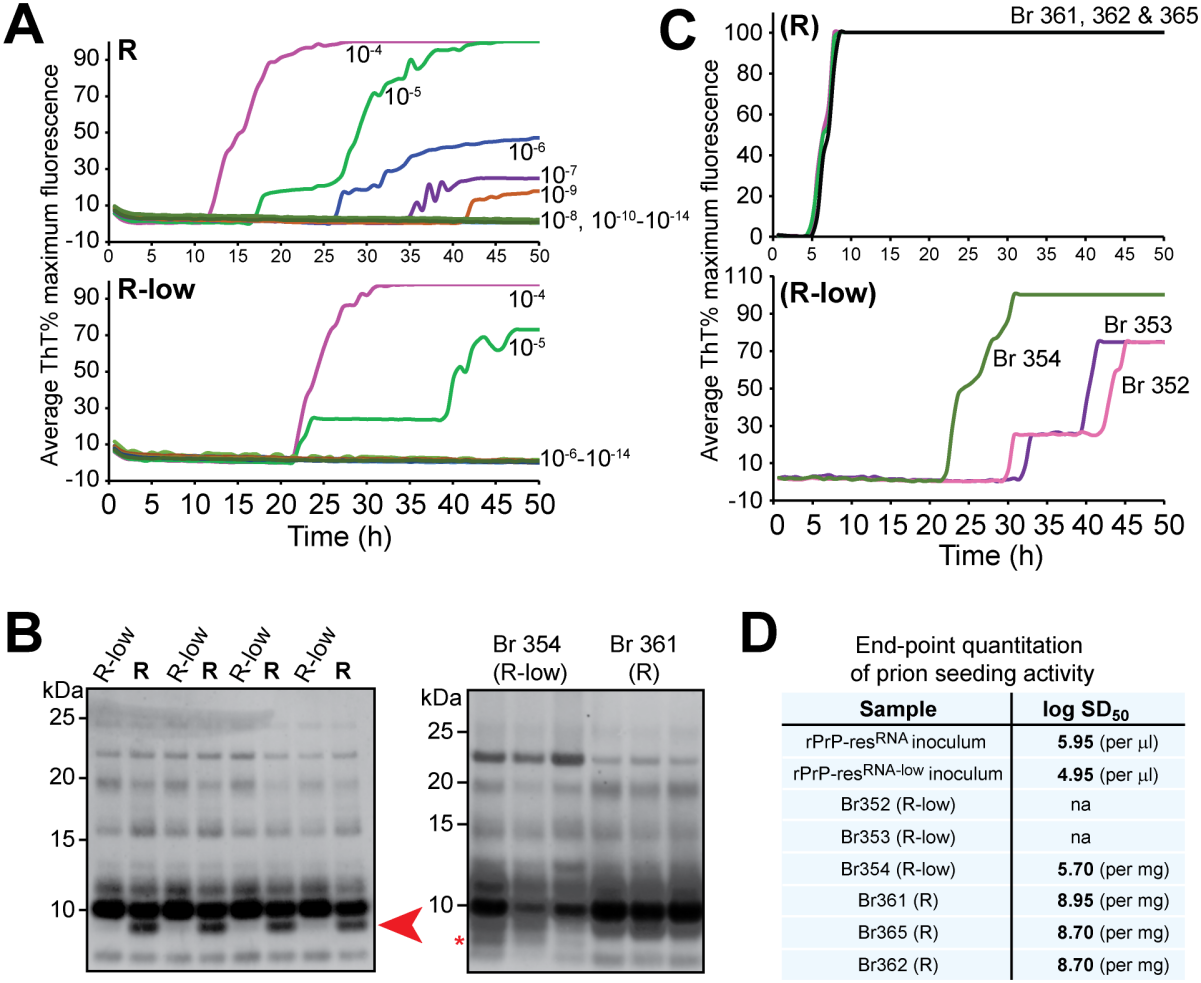
RT-QuIC detection of seeding activities in rPrP-res^RNA^, rPrP-res^RNA-low^ and brain homogenates from mice inoculated with rPrP-res^RNA^ or rPrP-res^RNA-low^ using Bank Vole rPrP. (**A**) rPrP-res^RNA^ (R, upper panel) and rPrP-res^RNA-low^ (R-low, lower panel) with designated dilutions were used to seed RT-QuIC reactions. (**B**) Western blots of PK digested RT-QuIC products seeded by rPrP-res^RNA^ (R) and rPrP-res^RNA-low^ (R-low) (Left panel) and by brain homogenates from mice 354 (inoculated with rPrP-res^RNA-low^) and 361 (inoculated with rPrP-res^RNA^) (Right panel). Each lane represents the RT-QuIC product collected from one single well. (**C**) RT-QuIC reactions were seeded with 10^−3^ brain tissue dilution from rPrP-res^RNA^ (Br 361, 362 and 365, upper panel) or rPrP-res^RNA-low^ (Br 352, 353 and 354, lower panel) inoculated mice. R and R-low in the parentheses indicate the inocula. (**D**) End-point quantitation of prion seeding activity in the original inocula (rPrP-res^RNA^ and rPrP-res^RNA-low^) and the brain homogenates of mice inoculated with rPrP-res^RNA^ (Br 361, 362 and 365) or rPrP-res^RNA-low^ (Br 352, 353 and 354). na, not available.

Both rPrP-res^RNA^ and rPrP-res^RNA-low^ were able to seed rPrP amyloid fibril growth in the RT-QuIC assay (Fig 8A). Since prion strain differences can be observed in the immunoblot banding profile of PK-digested RT-QuIC products[36], we subjected the rPrP-res^RNA^- or rPrP-res^RNA-low^-seeded RT-QuIC products to PK-digestion and western blot with the R20 antibody (recognizing residue 218–231 of hamster PrP). The PK-resistant banding patterns were almost identical except for a small PK-resistant band (Fig 8B, Left panel, indicated by an arrow), which is much more prominent in samples seeded by the pathogenic rPrP-res^RNA^. This result is consistent with the notion that rPrP-res^RNA^ and rPrP-res^RNA-low^ share a similar overall architecture, but have distinct small structural features.

Brain homogenates were prepared from three rPrP-res^RNA^-inoculated mice at terminal stage (rPrP-res^RNA^-BH) and three rPrP-res^RNA-low^-inoculated mice (Table 1). As expected, prion seeding activity was detected in rPrP-res^RNA^-BH (Fig 8C, upper panel). Surprisingly, positive RT-QuIC results were also obtained with brain homogenates prepared from all three mice inoculated with non-pathogenic rPrP-res^RNA-low^ (rPrP-res^RNA-low^-BH), which were without any signs of neurological dysfunction (Fig 8C, lower panel). To quantitate the total seeding activity, we performed end-point RT-QuIC quantitation[37] of the original inocula and the inoculated brain homogenates (Fig 8D). The log SD_50_ of the rPrP-res^RNA-low^ inoculum was 4.95 /μl, which gave a total SD_50_ of 10^6.25^ in the 20 μl inoculum (volume used to inject one mouse). Assuming that the total weight of a mouse brain is around 500 mg, the total SD_50_ in the brain of Br354 mouse was around 10^8.4^. The more than two orders of magnitude increase suggests that some amplification of the seeding activity occurred in the non-pathogenic rPrP-res^RNA-low^-inoculated brain. Consistent with this interpretation, seeding activity was detected in each of 5 mice receiving secondary transmission from a mouse (Br355) that was inoculated with the non-pathogenic rPrP-res^RNA-low^ (S10 Fig). Quantitative RT-QuIC comparisons of the Br355-derived inoculum and the brains of the second passage mice indicated ~1,700-fold increases in seeding activity in each case.

The brain homogenate seeded RT-QuIC products were also subject to PK-digestion and western blot analysis (Fig 8B, right panel). All of the PK-resistant bands detected in the RT-QuIC products seeded with rPrP-res^RNA^ were detected in the RT-QuIC products seeded by rPrP-res^RNA^-BH (Fig 8B), indicating a faithful *in vivo* propagation of the rPrP-res^RNA^ conformation. Although the non-pathogenic rPrP-res^RNA-low^-BH seeded RT-QuIC products also retained most of the PK-resistant bands, extra PK-resistant bands were also detected (Fig 8B, right panel, asterisk represents an example), which may reflect an adaptation of the non-pathogenic rPrP-res^RNA-low^ to the *in vivo* environment.

Together, these findings provided further support for the overall similarity between two distinct rPrP-res conformers. More importantly, the end-point analysis provided the first evidence that despite the drastic difference in pathogenicity, both rPrP-res conformers are active *in vivo*.

## Discussion

Our study revealed that although rPrP-res^RNA-low^ appears active and leads to the replication of prion seeding activity *in vivo*, it does not automatically result in pathogenic changes or the development of prion disease, at least for two consecutive passages in wild-type mice. The pathogenic and non-pathogenic rPrP-res conformers show many similarities in their overall architecture, suggesting that relatively small structural differences determine distinct biological properties of these rPrP-res aggregates. Moreover, our results support a critical role of cofactor in guiding the *de novo* rPrP-res formation, and suggest that different cofactors guide PrP misfolding in distinct manners, resulting in different rPrP-res conformers.

An interesting finding of this study is the ability of rPrP-res^RNA-low^ to cause the replication of seeding activity *in vivo*. This finding is reminiscent of previous reports that GSS patients’ brain homogenates containing only PrP amyloid fibrils, brain homogenates from diseased transgenic mice overexpressing P101L PrP, or synthetically generated rPrP amyloid fibrils are able to seed PrP amyloid plaque formation, but completely fail to cause any pathological changes of prion disease in P101L knock-in mice[38–40]. However, it has to be noted that we did not observe any PrP amyloid plaques in rPrP-res^RNA-low^ inoculated mouse brains. Together with the facts that wild-type mice were used in our analyses and that amyloid fibrils were likely only a part of rPrP-res (fibrils were only detected in certain fields with highly concentrated, PK-digested rPrP-res preparations), the rPrP-res^RNA-low^ likely propagates *in vivo* in a manner different from amyloid fibril seeding in P101L knock-in mice. Nevertheless, both lines of studies support that the *in vivo* PrP seeding does not necessarily lead to disease pathology.

The most likely reason for the difference in pathogenicity is the structural difference between rPrP-res^RNA^ and rPrP-res^RNA-low^. Interestingly, our results revealed a similar architecture (and thus likely similar overall folding motif) of the pathogenic rPrP-res^RNA^ and non-pathogenic rPrP-res^RNA-low^, which likely resulted from the strong influence of RNA and POPG cofactors on rPrP structure[41–43] that guides rPrP conversion. It appears that relatively minor structural differences between rPrP-res conformers are sufficient to result in large differences in *in vivo* pathogenicity. Understanding the nature of these structural differences requires studies employing higher resolution biophysical methods, and such future studies are of fundamental importance to understanding the molecular basis for prion infectivity.

The role of cofactor molecules in generating infectious prions is supported by many *in vitro* conversion studies[7,10,21,44–46], but the mechanisms are yet to be established. The highly pathogenic rPrP-res^RNA^ and rPrP-res^PE^ were formed with two different sets of cofactors, RNA+POPG[7] or PE only[10], respectively, and have displayed distinct prion strain properties in mice[21]. When PE was omitted from the sPMCA reaction, the propagation of rPrP-res^PE^ either stopped or led to the emergence of rPrP-res^protein-only^, which has a smaller PK-resistant core and no detectable *in vivo* pathogenicity[21]. Adding PE back to rPrP-res^protein-only^-seeded sPMCA reactions did not restore the rPrP-res^PE^ conformer[21]. The cofactor-dependence for rPrP-res^PE^ propagation supports a role of cofactor in rPrP conversion, but leaves open the question whether cofactors are mandatory for prion pathogenicity *in vivo*.

Our study demonstrates that cofactors are essential for the propagation of pathogenic rPrP-res^RNA^ and non-pathogenic rPrP-res^RNA-low^ or rPrP-res^NIH^. For the pathogenic rPrP-res^RNA^, omitting cofactors not only abolished the rPrP-res^RNA^ propagation but also eliminated the ability of the sPMCA products to convert endogenous PrP^C^ in CAD5 cells. Since exactly the same cofactors were required for the formation of the pathogenic rPrP-res^RNA^, and the non-pathogenic rPrP-res^RNA-low^ or rPrP-res^NIH^, our results demonstrated that it is not the cofactors, but rather the distinctive structural features of rPrP-res^RNA^ that determine the pathogenicity.

In this study, no “protein-only”, self-propagating, rPrP-res conformer was formed in the absence of cofactors in either rPrP-res^RNA^- or rPrP-res^RNA-low^-seeded sPMCA reactions, which is different from those seeded by rPrP-res^PE^[21]. Many factors may account for this difference, but the difference between the two sets of cofactors is likely to be the key. Full-length rPrP has a high isoelectric point (pI > 9) and binds to negatively charged RNA and POPG[41,42]. We and other groups have shown that the binding of rPrP to anionic lipids or RNA results in significant rPrP conformational changes[41–43,47]. PE, on the other hand, is a neutral phospholipid that has little or no *in vitro* interaction with rPrP[47]. The sonication in sPMCA may foster a unique rPrP-PE interaction leading to the formation of PE-dependent rPrP-res^PE^. Thus, both rPrP-res^PE^ and rPrP-res^RNA^ can be propagated in sPMCA, but likely through different pathways, which is in agreement with their different strain properties *in vivo*[21]. Consistent with the idea that different cofactors used in sPMCA may lead to different rPrP conformations, the non-pathogenic rPrP-res forms derived from two sPMCA reactions, rPrP-res^RNA-low^ and rPrP-res^protein-only^, have drastic differences in cofactor dependence in their propagation, which strongly indicates a difference in their structures.

Even though native prions are generally not present with fibrillar structure *in vivo*, diseased brain homogenates do have the ability to seed rPrP amyloid fiber formation[29–31,48], and this property has been successfully developed into an ultrasensitive diagnostic tool[33]. Our data showed that, akin to brain-derived prions, all three cofactor-dependent rPrP-res conformers studied are able to seed rPrP amyloid fibril growth, supporting the structural similarity among those conformers. The non-pathogenic forms (i.e. rPrP-res^RNA-low^ and rPrP-res^NIH^) have apparently stronger seeding capacity than the pathogenic rPrP-res^RNA^ in the amyloid fibril formation assay, which may reflect the fact that the non-pathogenic rPrP-res^RNA-low^ maintains more seeding competent conformation in the presence of 2M GdnHCl, a semi-denaturing buffer system used for growing rPrP amyloid fibrils[32]. However, using the non-denaturing conditions of RT-QuIC, the seeding activity of the non-pathogenic form was about a log lower than the pathogenic form. This again suggests differences in the conformation and seeding capabilities of these two conformers.

In summary, our current study provides evidence that (i) cofactors are able to guide rPrP misfolding to result in different rPrP-res conformers, (ii) unique structural features of the rPrP-res determine the pathogenicity, and (iii) the *in vivo* rPrP-res seeding activity is not necessarily equal to disease pathogenicity. These novel insights help to elucidate the molecular basis for prion infectivity.

## Materials and methods

### Generation of rPrP-res

Recombinant murine PrP 23–230 purification and sPMCA experiments were performed as previously described[7,19,45,49]. For seeded sPMCA, 10 μL of rPrP-res^RNA^ seed was added to the substrate and the mixture was subjected to 1 round of PMCA. After each round, 10 μL of the PMCA product was transferred to a new tube containing 90 μL of substrate for another round. For unseeded PMCA, the same protocol was followed except that 10 μL of PBS instead of rPrP-res^RNA^ was added to the first tube of substrate. For the cofactor-free sPMCA, mouse liver total RNA and synthetic phospholipid POPG were omitted during substrate preparation. To detect the generation of rPrP-res, 10 μL of PMCA product was incubated with 10 μL PK (100 μg/mL unless stated otherwise) for 30 min at 37°C followed by the addition of 2 mM PMSF. The PK-digested samples were subjected to SDS-PAGE and western blotting. All the PK-resistant PrP fragments were detected using POM1 primary anti-PrP antibody[50] unless stated otherwise.

### The enzyme-linked immunospot (Elispot) cell infection assay

The Elispot cell infection assay was adapted from previous studies[22,23] with minor adjustments. Briefly, 200 μL of PMCA products at round 6 were collected and centrifuged at 100,000 x *g* for 1 h at 4°C. The pellets were then washed twice, with centrifugation at 100,000 x *g* for 1 h at 4°C after each wash. After the final wash, the pellets were resuspended in 200 μL of CAD5 growth media (OPTI-MEM, 5% BGS, and 1% penicillin and streptomycin) and sonicated for 30 sec with 50% output using a Misonic Sonicator (XL2020). Then each sample was serially diluted 10, 100, and 1,000 times, and 60 μL of undiluted and diluted samples were used to infect CAD5 cells. After two 1:10 splits, 20,000 CAD5 cells/well were transferred to the Millipore 96-well Elispot plates (MSIPN4W) and subjected to the Elispot assay. The images were taken by S6 Micro Analyzer (CTL Analyzers, LLC) and processed by the ImmunoSpot software (CTL Analyzers, LLC). The graph was generated using GraphPad Prism (GraphPad Software, Inc.). To validate the Elispot data, the remaining infected cells were lysed and subjected to PK digestion (100 μg/mL PK, 37°C, 30 min) and SDS-PAGE. The PK-resistant PrP fragments were detected by western blots using POM1 anti-PrP antibody.

### Mouse bioassays

The mouse bioassays were performed as previously described[7,19,45,49]. In brief, 20 μL of purified rPrP-res was inoculated into a mouse intracerebrally. Second-round transmission, animal monitoring, biochemical analyses, and histopathological analyses were performed as previously described[7,19,45,49].

### Ethics statement

This study was carried out in accordance with the recommendations in the Guide for the Care and Use of Laboratory Animals of the National Institutes of Health. The protocols were approved by the Institutional Animal Care and Use Committees of the Van Andel Research Institute (Assurance Number A4383-01).

### Immunoprecipitation with anti-PrP^Sc^ conformational antibodies

Protein-G DynalBeads (100 μL; Life Technologies) were washed twice with 250 μL of coating buffer (0.1% BSA in PBS), incubated in 250 μL of coating buffer at 4°C overnight, and then resuspended in 300 μL of coating buffer for use. The rPrP-res-seeded PMCA products containing 100 ng of PrP were incubated with no antibody or with 2.5 μg of conformational anti-PrP^Sc^ antibodies in 250 μL of incubation buffer (10 mM Tris, 150 mM NaCl, 0.28% Triton X-100, pH 7.5) at 4°C overnight, then mixed with 30 μL of resuspended coated beads and incubated at room temperature for 4 h, followed by washing 3 times with 100 μL of incubation buffer. The beads were then resuspended in 20 μL of SDS-PAGE sample buffer, boiled for 10 min, and subjected to SDS-PAGE and western blotting for detection of PrP using the anti-PrP M-20 polyclonal antibody.

### Conformational stability assay

The conformational stability assay was performed as previously described[28]. Briefly, aliquot of rPrP-res^RNA^, rPrP-res^RNA-low^ or rPrP-res^NIH^ was mixed with an equal volume of GdnHCl solutions to reach final concentrations of 0, 0.5, 1.0, 1.5, 2.0, 2.5, 3.0, 3.5, and 4.0 M and kept at 37°C for 1 h, followed by centrifugation at 20,000 x g for 1 h at 22°C. Supernatants were removed and pellets were resuspended in SDS-PAGE sample buffer and subjected to SDS-PAGE and western blotting for detecting PrP using the anti-PrP POM1 antibody.

Western blotting images were obtained with Fujifilm LAS-4000 imaging system and banding intensity was quantified with ImageJ. The denaturation curves for each rPrP-res conformer were generated by fitting the insoluble PrP (%) as a function of GdnHCl concentrations using a Sigmoidal, 4 parameter logistic equation in GraphPad Prism.

### Amyloid fibril formation assay

Amyloid fibril formation was performed as previously described[51]. For unseeded growth, 0.5 mg/mL of rPrP was incubated in 2 M guanidine hydrochloride (GdnHCl), 100 mM potassium phosphate buffer, pH 6.5, and 20 μM Thioflavin T (ThT). The reaction volume was 200 μL per well in 96-well plates (Corning, Lot No. 065514030). In seeded reactions, 1 μL of preformed mouse rPrP fibrils (0.5 mg/mL) or 5 μL of treated rPrP-res was added to each well. The plate was incubated at 37°C with continuous shaking on a microplate reader (SYNERGY2, BioTek). The fibril kinetics was monitored by measuring ThT fluorescence intensity every 15 min using 440-nm excitation and 480-nm emission. The amyloid-formation kinetics was generated using GraphPad Prism (GraphPad Software, Inc.). The lag time was determined when the ThT fluorescence reached threefold above the baseline[52].

The sPMCA products of each rPrP-res conformer were digested with Benzonase and PK and purified as previously described[20] to seed amyloid fibril formation.

### Atomic force microscopy imaging and substrate preparation

Atomic force microscopy (AFM) images were collected on a Multimode 8 AFM fitted with the Nanoscope V controller (Bruker Co., USA). Images were acquired in ScanAsyst mode using silicon tips. Samples were absorbed on freshly cleaved mica and then rinsed with nanopure water and dried with compressed air. Images were analyzed using Scanning Probe Image Processor (SPIP) software (version 6.5.2, Image Metrology A/S, Lyngby, Denmark) or NanoScope Analysis 1.5 software (Bruker Co., USA).

For imaging sPMCA products, rPrP-res^RNA^ or rPrP-res^RNA-low^ was treated with Benzonase (200 U/mL, 1 mM MgCl2) plus α-amylase (Sigma, 5 units/mL) at 37°C overnight, followed by PK digestion (25 μg/mL) at 37°C for 30 minutes. The treated samples were centrifuged at 100,000 x *g* for 1 h at 4°C and the pellets were washed once in 10 mM Potassium Phosphate buffer (pH 7.4). The control sample, containing all the cofactors but no rPrP, went through the same treatments except the PK-digestion. The final pellets were resuspended in appropriate volumes of ddH_2_O for imaging. For imaging rPrP amyloid fibrils, samples were centrifuged at 100,000 x *g* for 1 h at 4°C. The pellets were washed twice with ddH_2_O and resuspended in appropriate volumes of ddH_2_O for imaging.

### RT-QuIC

#### Protein purification

The Bank Vole recombinant prion protein (rPrP^Sen^) (residues 23 to 230; Methionine at residue 109; accession no. AF367624) was purified as previously described[53]. The vectors were transformed into Rosetta (DE3) *Escherichia coli* and grown in Luria broth medium with the addition of kanamycin and chloramphenicol. The expression of the protein was induced using the autoinduction system[54,55]. Inclusion bodies containing the protein were denatured and the protein was isolated using Ni-nitrilotriacetic acid (NTA) superflow resin (Qiagen) with an ÄKTA fast protein liquid chromatographer (GE Healthcare Life Sciences). Refolding of the protein was done on the column by means of a guanidine HCl reduction gradient. Elution was done with an imidazole gradient as described. Following elution, the protein was extensively dialyzed into 10 mM sodium phosphate buffer (pH 5.8), filtered (0.22-μm syringe filter (Fisher)) and stored at -80°C in 1mL aliquots. The protein concentration was determined by absorbance at 280 nm.

#### Brain homogenate and PMCA product dilution for RT-QuIC

Brain homogenates (BH; 10% w/v) were prepared as previously described[37] and stored at -80°C. For RT-QuIC analysis, BHs or PMCA products were serially diluted in 0.1% SDS (sodium dodecyl sulfate, Sigma)/N2 (Gibco)/PBS as previously reported[36] with the last dilutions prepared to a final concentration of 0.05% SDS/N2/PBS.

#### RT-QuIC assay protocol

RT-QuIC analysis was performed as previously reported[37]. The reaction mix included 10 mM phosphate buffer (pH 7.4), 300 mM NaCl, 0.1 mg/ml rPrP, 10 μM Thioflavin T (ThT), 1 mM ethylenediaminetetraacetic acid tetrasodium salt (EDTA), and 0.001% SDS. Aliquots of the reaction mix (98 μL) were loaded into each well of a black 96-well plate with a clear bottom (Nunc). Reactions were seeded with 2 μL of indicated BH or PMCA product dilutions. Following sealing with a plate sealer film (Nalgene Nunc International), the plate was incubated at 42°C in a BMG FLUOstar Omega plate reader with cycles of 1 min shaking (700 rpm double orbital) and 1 min rest. ThT fluorescence measurements (450 +/-10 nm excitation and 480 +/-10 nm emission; bottom read) were taken every 45 min.

To compensate for minor differences in baselines between fluorescent plate readers and across several experiments, the data was normalized to a percentage of the maximal fluorescence response (260,000 rfu) of the plate readers following subtraction of the baseline, as described[33]. ThT fluorescence was plotted versus reaction time (hours). The reactions were classified as RT-QuIC positive base on criteria similar to those previously described for RT-QuIC analyses of brain specimens[33,37].

#### Immunoblotting of proteinase K (PK) digested RT-QuIC products

The RT-QuIC reaction conversion products were collected from the wells by extensive and vigorous scraping followed by pipetting. The samples were treated with 10 μg/ml Proteinase K (PK) for 1 hour at 37°C with 400 rpm continuous orbital shaking. Equal volumes of PK-treated reactions were run on 12% Bis-Tris NuPAGE gels (Invitrogen). The proteins were transferred to an Immobilon P membrane (Millipore) using the iBlot Gel Transfer System (Invitrogen). The membranes were incubated with R20 primary antiserum (hamster epitope: residues 218–231)[56] diluted 1:15,000, followed by goat-anti-rabbit secondary antibody dilutes 1:5000, and visualized with the Attophos AP fluorescent substrate system (Promega) according to the manufacturer's recommendations.

### Statistical analysis

Means are presented with their standard deviations and compared by one-way analysis of variance (ANOVA) followed by Tukey’s multiple comparisons test, two-way ANOVA analyses followed by Tukey’s multiple comparisons test, or by two-tailed unpaired multiple t test with Holm-Sidak’s correction. Statistical analysis was performed with GraphPad Prism 6.05.

## Supporting information

S1 Fig Generation of rPrP-res^RNA-low^. Generation of rPrP-res^RNA-low^ in the presence of mouse liver total RNA and synthetic phospholipid POPG in unseeded sPMCA reactions. Immunoblot analyses of PK-digested sPMCA products from four representative reactions revealed the *de novo* generation of rPrP-res^RNA-low^. C, undigested rPrP as a control. The protocol is exactly the same as that for the *de novo* generation of the pathogenic rPrP-res^RNA^. The opportunity to generate the non-pathogenic rPrP-res^RNA-low^ is higher than that of rPrP-res^RNA^ in this reaction system.
(TIF)

S2 Fig Evaluation of spongiosis. Representative images of Hematoxylin and Eosin (H&E) stain of brain sections prepared from mice receiving intracerebral inoculation of rPrP-res^RNA-low^ (1^st^ transmission), second round transmission (2^nd^ transmission), and age-matched control mice as indicated. No spongiosis was detected.
(TIF)

S3 Fig Evaluation of astriogliosis. Representative images of brain sections prepared from mice receiving intracerebral inoculation of rPrP-res^RNA-low^ (1^st^ transmission), second round transmission (2^nd^ transmission), and age-matched control mice that were stained with an antibody against glial fibrillary acidic protein (GFAP). No difference between control and experimental animals was observed.
(TIF)

S4 Fig Evaluation of aberrant PrP deposition. Representative images of brain sections prepared from mice receiving intracerebral inoculation of rPrP-res^RNA-low^ and age-matched control mice that were stained with SAF84 anti-PrP antibody. No aberrantly deposited PrP was detected.
(TIF)

S5 Fig Evaluation of PK-resistant PrP deposition. Paraffin-embedded tissue (PET) blot analysis of mouse brains receiving intracerebral inoculation of rPrP-res^RNA-low^ (1^st^ transmission), second round transmission (2^nd^ transmission), and an age-matched control mouse brain. No PK-resistant PrP was detected. A positive control of rPrP-res^RNA^ inoculated mouse brain was included to demonstrate that the PET blot protocol is able to detect PK-resistant PrP.
(TIF)

S6 Fig PK-resistant PrP in rPrP-res^RNA^ or rPrP-res^RNA-low^ infected CAD5 cells. Validation of Elispot cell infection assay results (Fig 1C) by western blots of PK digested cell lysates. * indicates the rPrP-res^RNA^ (**R**) applied to CAD5 cells; ** indicates the rPrP-res^RNA-low^ (**R-low**) applied to CAD5 cells; C, undigested naïve CAD5 cell lysates as controls. PrP was detected with POM1 anti-PrP antibody.
(TIF)

S7 Fig Validation of Elispot cell infection assay results by western blots of PK digested cell lysates. * indicates rPrP-res^RNA^ applied to CAD5 cells; C1, undigested naïve CAD5 cell lysates as a control; C2, PK digested naïve CAD5 cell lysates as a control. PrP was detected with POM1 anti-PrP antibody.
(TIF)

S8 Fig Cell infection assay of rPrP-res^RNA^, rPrP-res^RNA-low^, or rPrP-res^NIH^. (**A**) Elispot assay results. (**B**) PK-digestion of CAD5 cells infected with rPrP-res^RNA^ (**R**), rPrP-res^RNA-low^ (**R-low**), or rPrP-res^NIH^ (**NIH**) as indicated. * indicates rPrP-res^RNA^ applied to CAD5 cells; C1, undigested naïve CAD5 cell lysates as a control; C2, PK digested naïve CAD5 cell lysates as a control. PrP was detected with POM1 anti-PrP antibody.
(TIF)

S9 Fig Cell infection assay of rPrP amyloid fibers. (**A**) Elispot assay results. (**B**) PK-digested cell lysates of CAD5 cells infected with rPrP amyloid fibrils from Fig 6. CAD5 cells infected with rPrP-res^RNA^ (**R**) were included as a positive control. C1, undigested naïve CAD5 cell lysates as a control; C2, PK digested naïve CAD5 cell lysates as a control. PrP was detected with POM1 anti-PrP antibody.
(TIF)

S10 Fig RT-QuIC assay of brain lysates prepared from rPrP-res^RNA-low^ inoculated Br355 mouse and all mice received second round transmission. RT-QuIC reactions were seeded with indicated brain tissue dilution from rPrP-res^RNA-low^-inoculated Br355 mouse (the healthy mouse that was sacrificed at 399 dpi to prepare brain homogenate for second round transmission) and mice received second round transmission.
(TIF)

S1 Appendix Banding intensities of each conformational stability assay western blots (R, n = 3; R-low, n = 7; NIH, n = 5). The intensities at different concentrations of GdnHCl were quantified with ImageJ and presented in percentages of the un-denatured. Data in this spreadsheet was used to plot the denaturation curves shown in Figs 5B and 6D, and to analyze the statistical significances in GraphPad Prism.
(XLSX)
