# Supplementary figures and images for: Self-propagating, protease-resistant, recombinant prion protein conformers with or without in vivo pathogenicity
Source: PLoS Pathog. 2017 Jul 12;13(7):e1006491. doi: 10.1371/journal.ppat.1006491 (PMC5524416; doi:10.1371/journal.ppat.1006491)

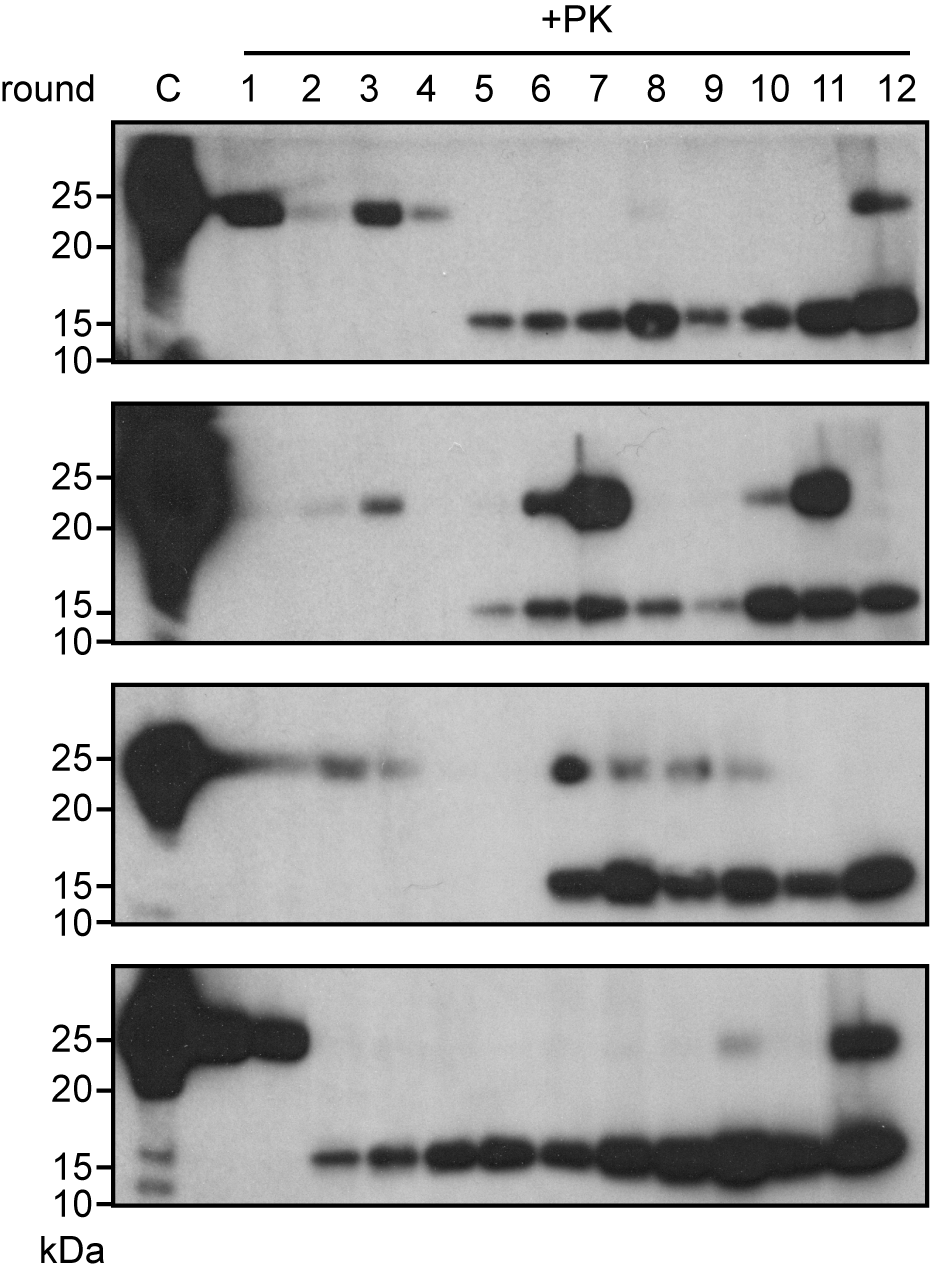

Supplement: S1 Fig — Generation of rPrP-resRNA-low in the presence of mouse liver total RNA and synthetic phospholipid POPG in unseeded sPMCA reactions. Immunoblot analyses of PK-digested sPMCA products from four representative reactions revealed the de novo generation of rPrP-resRNA-low. C, undigested rPrP as a control. The protocol is exactly the same as that for the de novo generation of the pathogenic rPrP-resRNA. The opportunity to generate the non-pathogenic rPrP-resRNA-low is higher than that of rPrP-resRNA in this reaction system. (TIF) [file ppat.1006491.s001.tif]

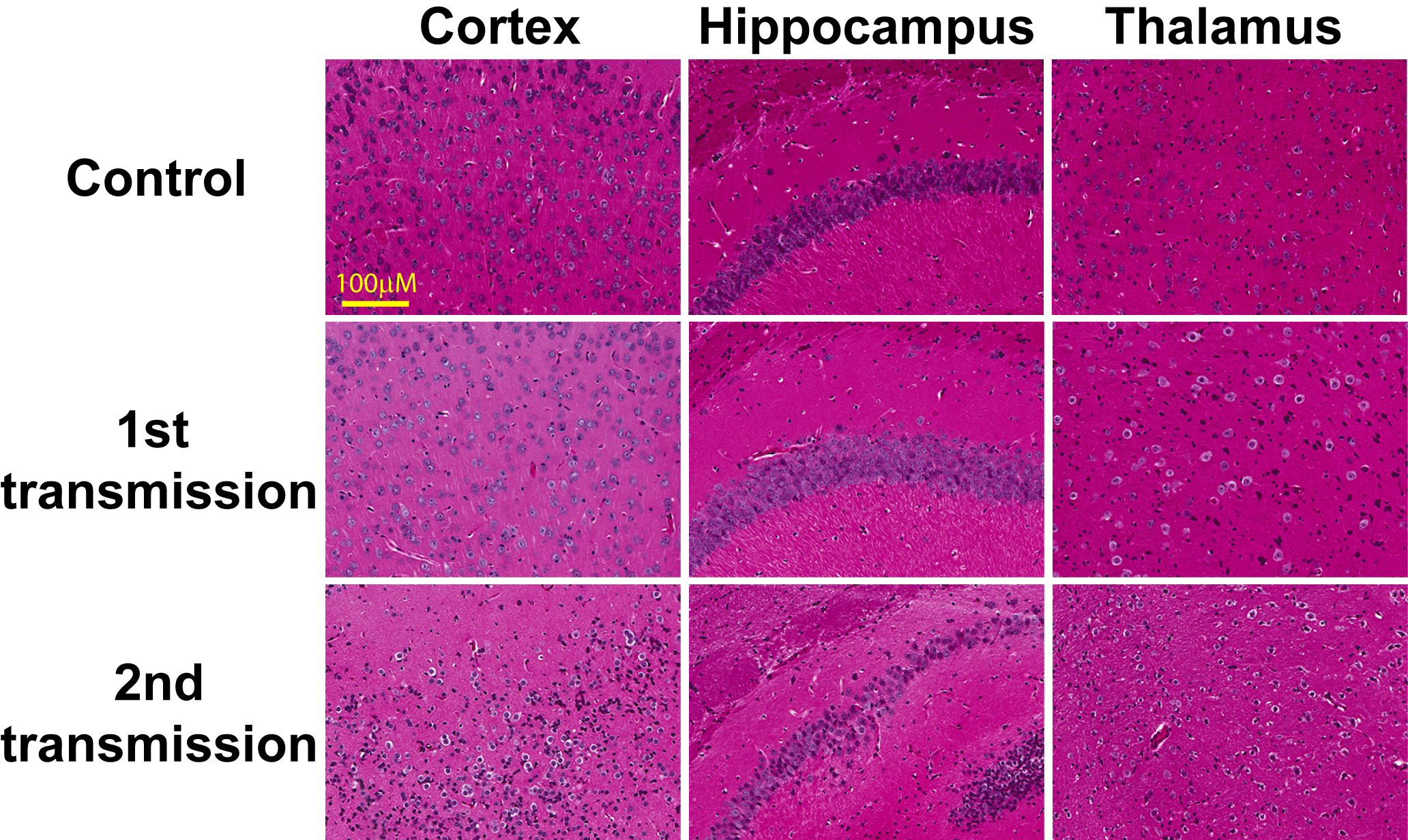

Supplement: S2 Fig — Representative images of Hematoxylin and Eosin (H&E) stain of brain sections prepared from mice receiving intracerebral inoculation of rPrP-resRNA-low (1st transmission), second round transmission (2nd transmission), and age-matched control mice as indicated. No spongiosis was detected. (TIF) [file ppat.1006491.s002.tif]

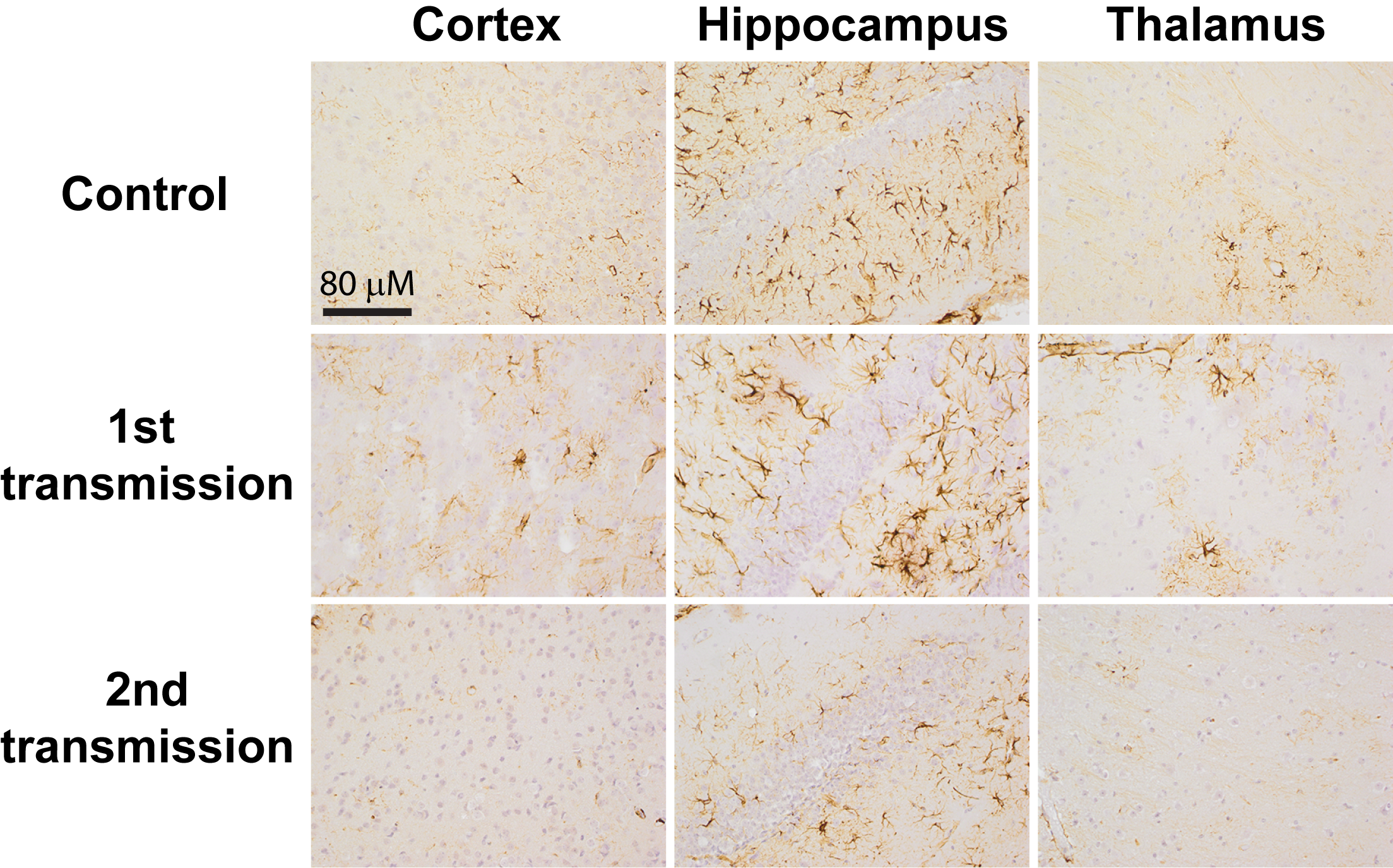

Supplement: S3 Fig — Representative images of brain sections prepared from mice receiving intracerebral inoculation of rPrP-resRNA-low (1st transmission), second round transmission (2nd transmission), and age-matched control mice that were stained with an antibody against glial fibrillary acidic protein (GFAP). No difference between control and experimental animals was observed. (TIF) [file ppat.1006491.s003.tif]

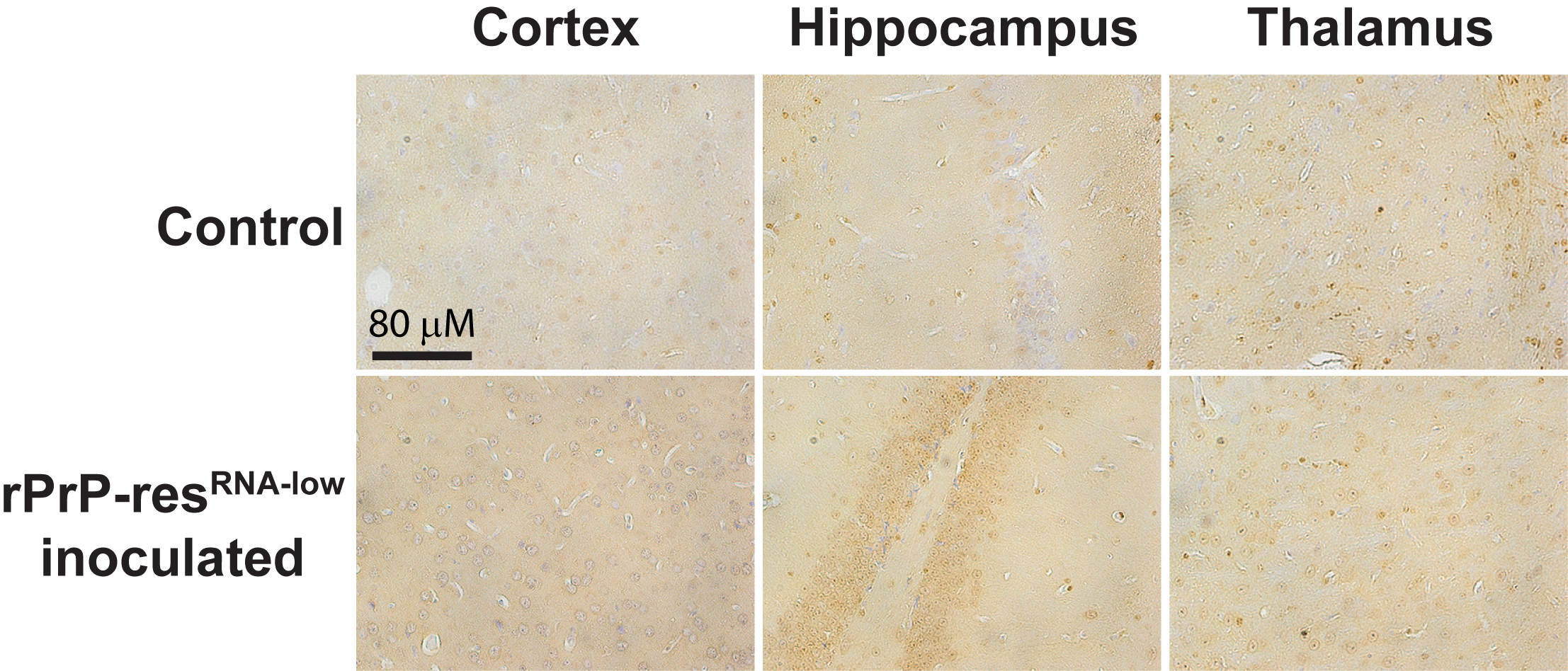

Supplement: S4 Fig — Representative images of brain sections prepared from mice receiving intracerebral inoculation of rPrP-resRNA-low and age-matched control mice that were stained with SAF84 anti-PrP antibody. No aberrantly deposited PrP was detected. (TIF) [file ppat.1006491.s004.tif]

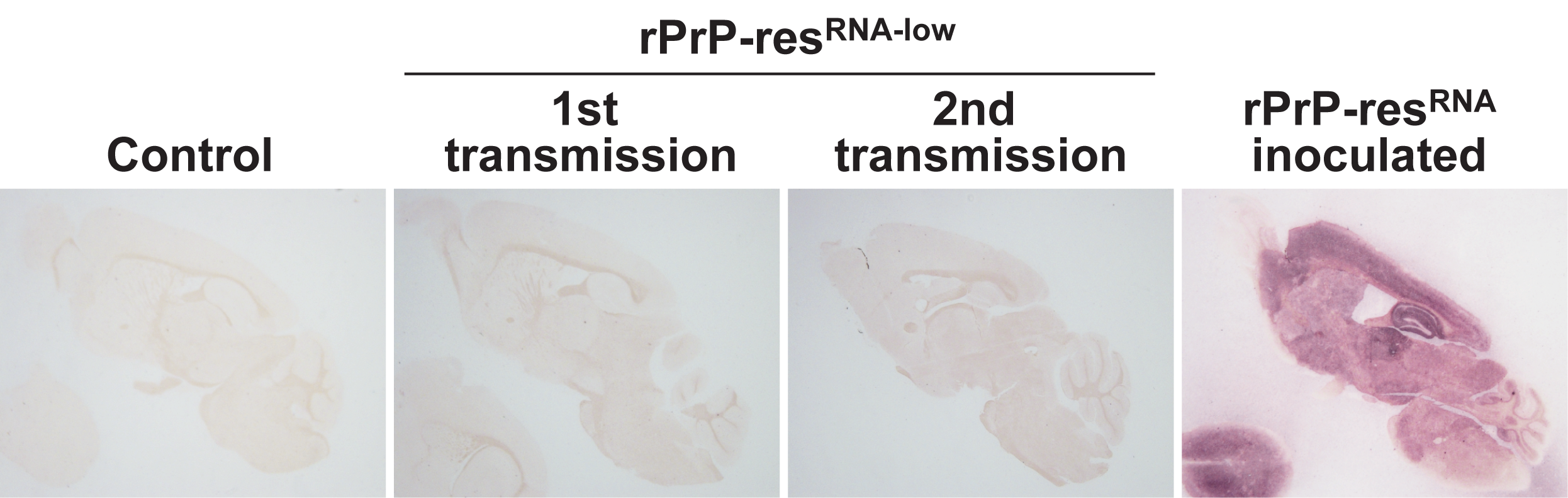

Supplement: S5 Fig — Paraffin-embedded tissue (PET) blot analysis of mouse brains receiving intracerebral inoculation of rPrP-resRNA-low (1st transmission), second round transmission (2nd transmission), and an age-matched control mouse brain. No PK-resistant PrP was detected. A positive control of rPrP-resRNA inoculated mouse brain was included to demonstrate that the PET blot protocol is able to detect PK-resistant PrP. (TIF) [file ppat.1006491.s005.tif]

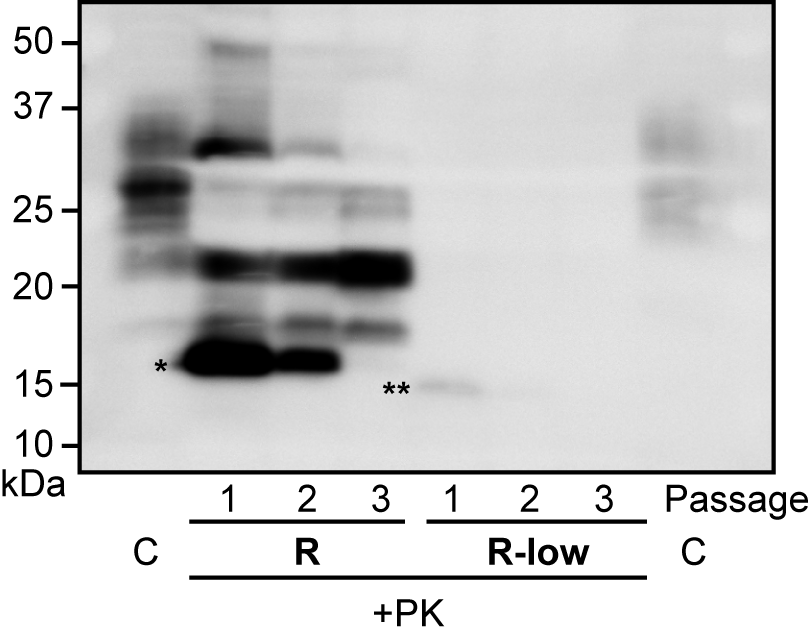

Supplement: S6 Fig — Validation of Elispot cell infection assay results (Fig 1C) by western blots of PK digested cell lysates. * indicates the rPrP-resRNA (R) applied to CAD5 cells; ** indicates the rPrP-resRNA-low (R-low) applied to CAD5 cells; C, undigested naïve CAD5 cell lysates as controls. PrP was detected with POM1 anti-PrP antibody. (TIF) [file ppat.1006491.s006.tif]

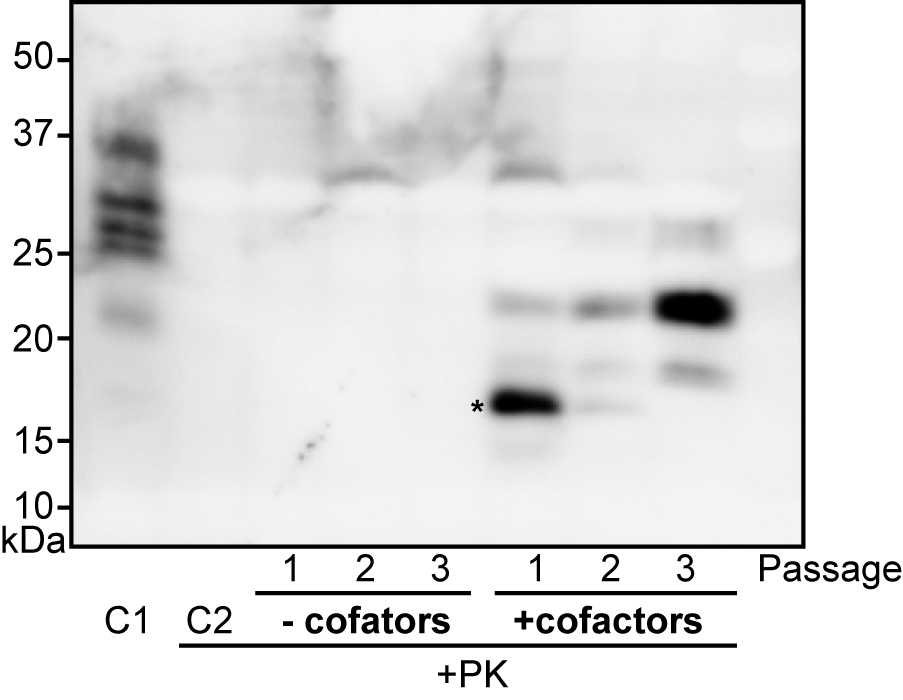

Supplement: S7 Fig — * indicates rPrP-resRNA applied to CAD5 cells; C1, undigested naïve CAD5 cell lysates as a control; C2, PK digested naïve CAD5 cell lysates as a control. PrP was detected with POM1 anti-PrP antibody. (TIF) [file ppat.1006491.s007.tif]

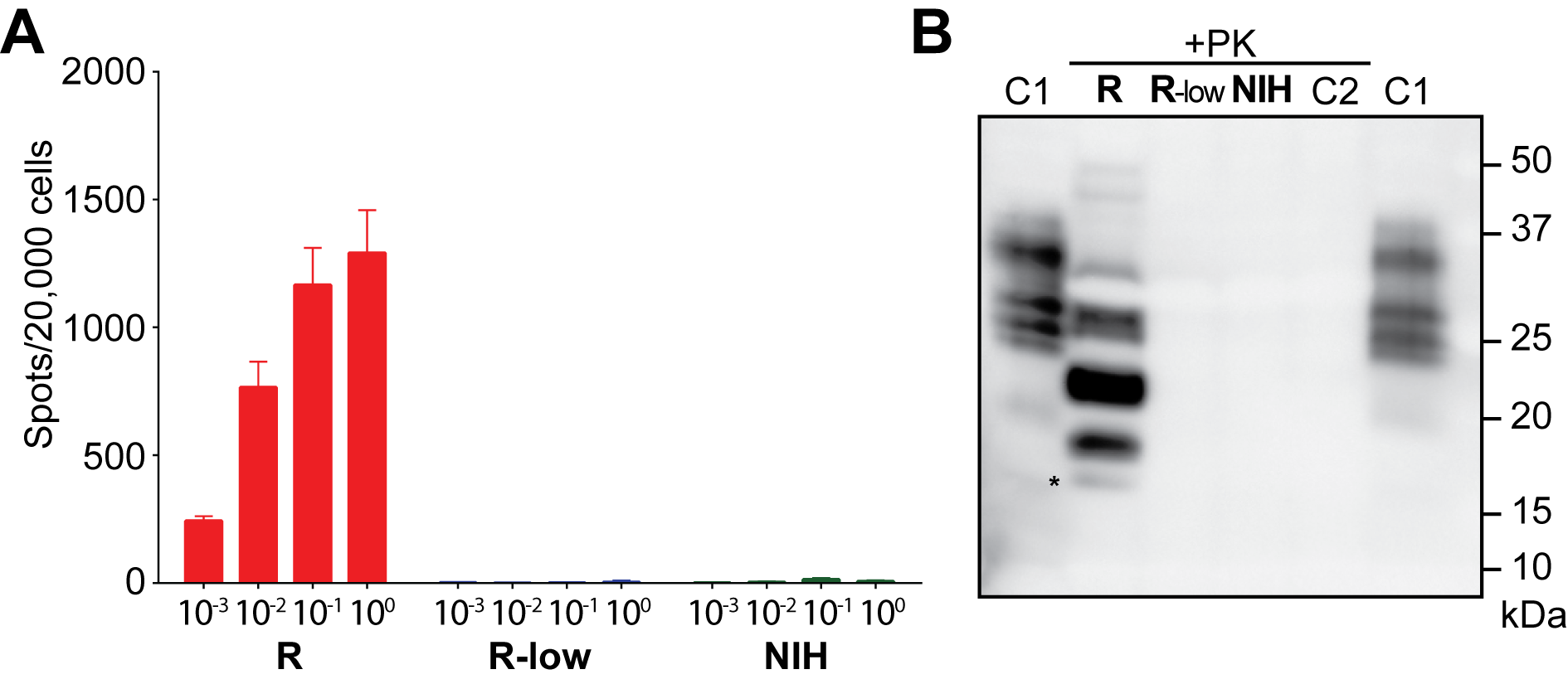

Supplement: S8 Fig — (A) Elispot assay results. (B) PK-digestion of CAD5 cells infected with rPrP-resRNA (R), rPrP-resRNA-low (R-low), or rPrP-resNIH (NIH) as indicated. * indicates rPrP-resRNA applied to CAD5 cells; C1, undigested naïve CAD5 cell lysates as a control; C2, PK digested naïve CAD5 cell lysates as a control. PrP was detected with POM1 anti-PrP antibody. (TIF) [file ppat.1006491.s008.tif]

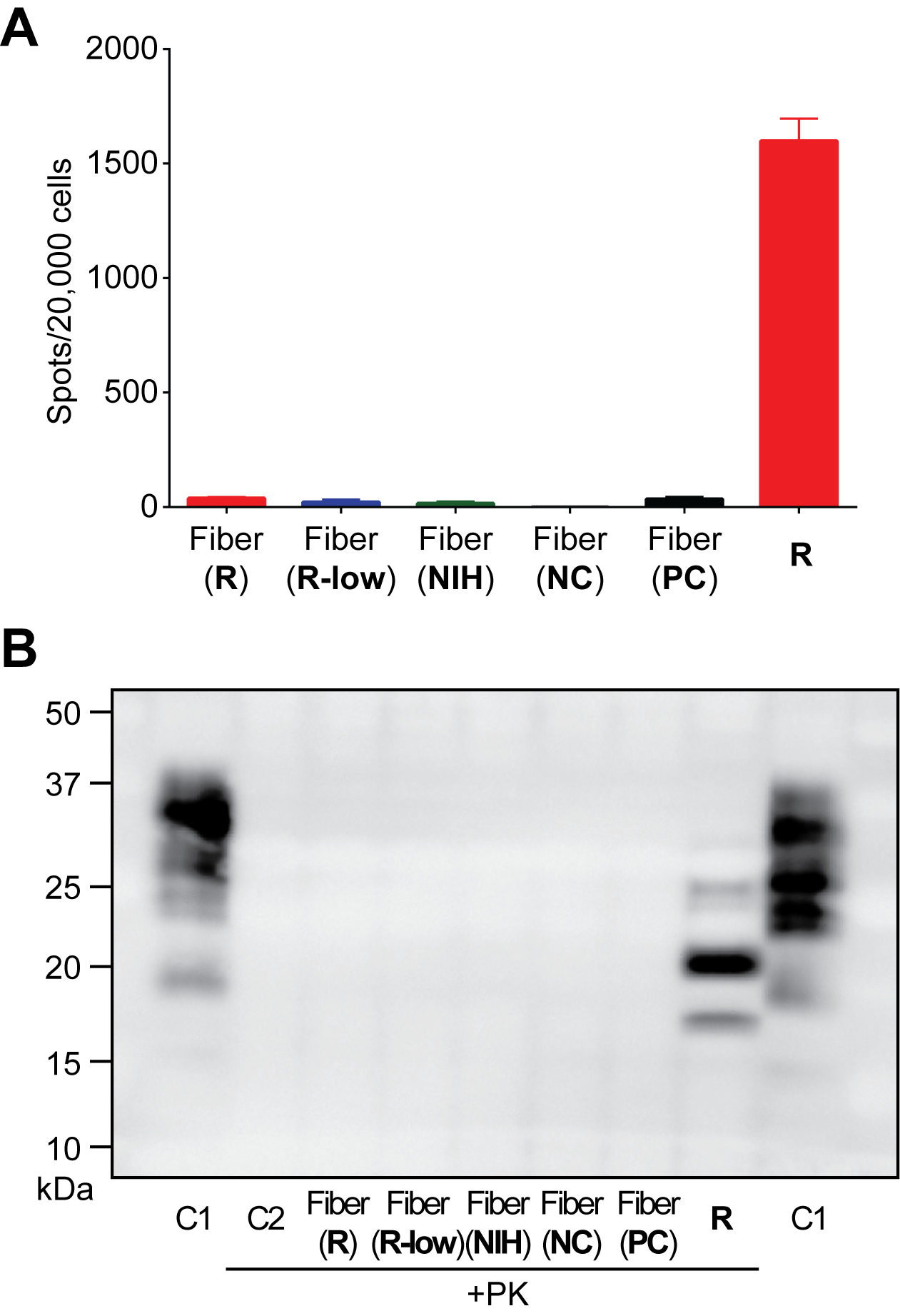

Supplement: S9 Fig — (A) Elispot assay results. (B) PK-digested cell lysates of CAD5 cells infected with rPrP amyloid fibrils from Fig 6. CAD5 cells infected with rPrP-resRNA (R) were included as a positive control. C1, undigested naïve CAD5 cell lysates as a control; C2, PK digested naïve CAD5 cell lysates as a control. PrP was detected with POM1 anti-PrP antibody. (TIF) [file ppat.1006491.s009.tif]

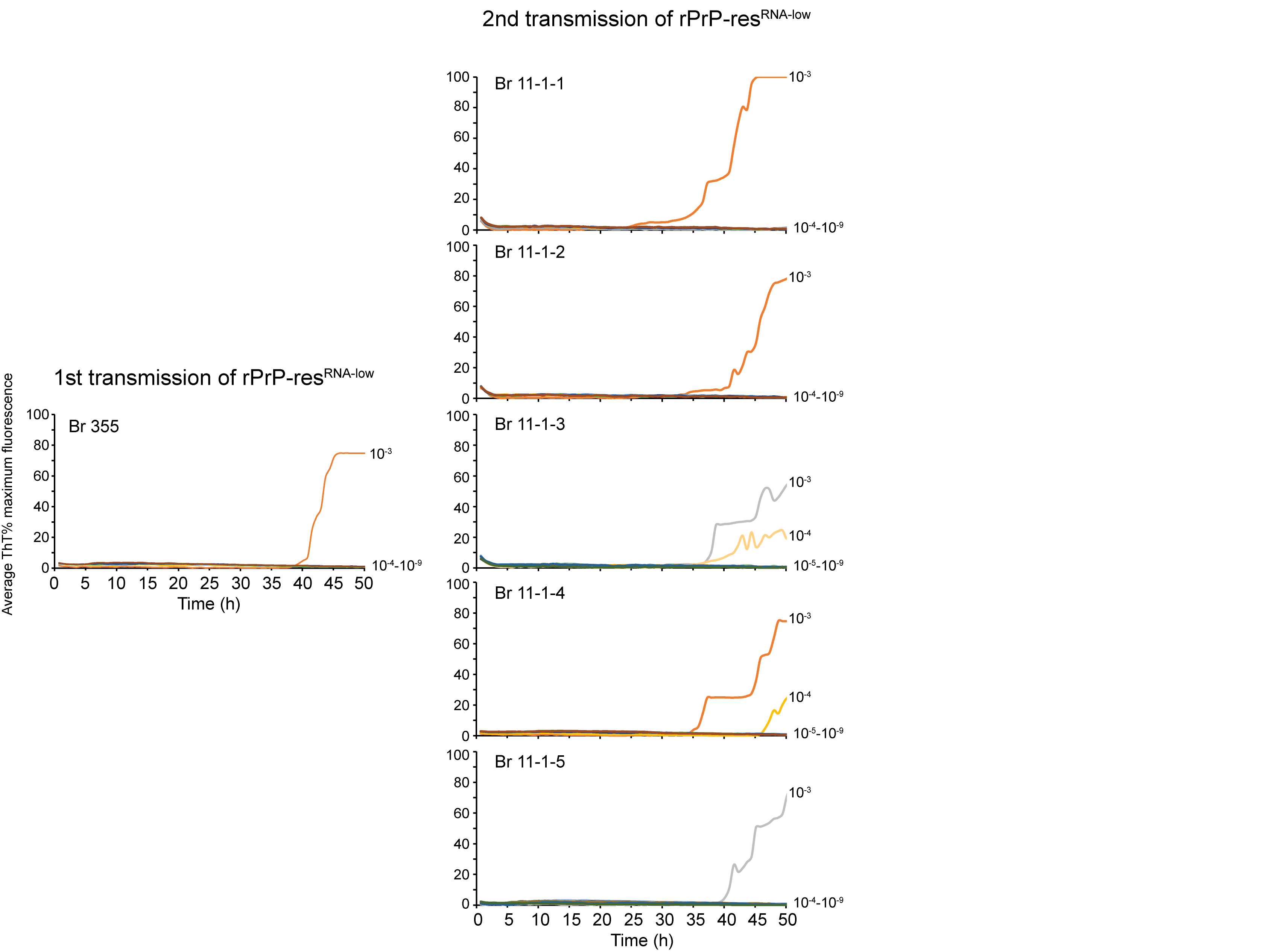

Supplement: S10 Fig — RT-QuIC reactions were seeded with indicated brain tissue dilution from rPrP-resRNA-low-inoculated Br355 mouse (the healthy mouse that was sacrificed at 399 dpi to prepare brain homogenate for second round transmission) and mice received second round transmission. (TIF) [file ppat.1006491.s010.tif]
